# Supplementary material for: Meta-analyses of Culex blood-meals indicates strong regional effect on feeding patterns
Source: PLoS Negl Trop Dis. 2025 Jan 24;19(1):e0012245. doi: 10.1371/journal.pntd.0012245 (PMC11785302; doi:10.1371/journal.pntd.0012245)
Supplement: S1 Fig — PRISMA flowchart of selection and inclusion process for the meta-analysis of Culex blood-meal studies. (DOCX) [file pntd.0012245.s003.docx]

*
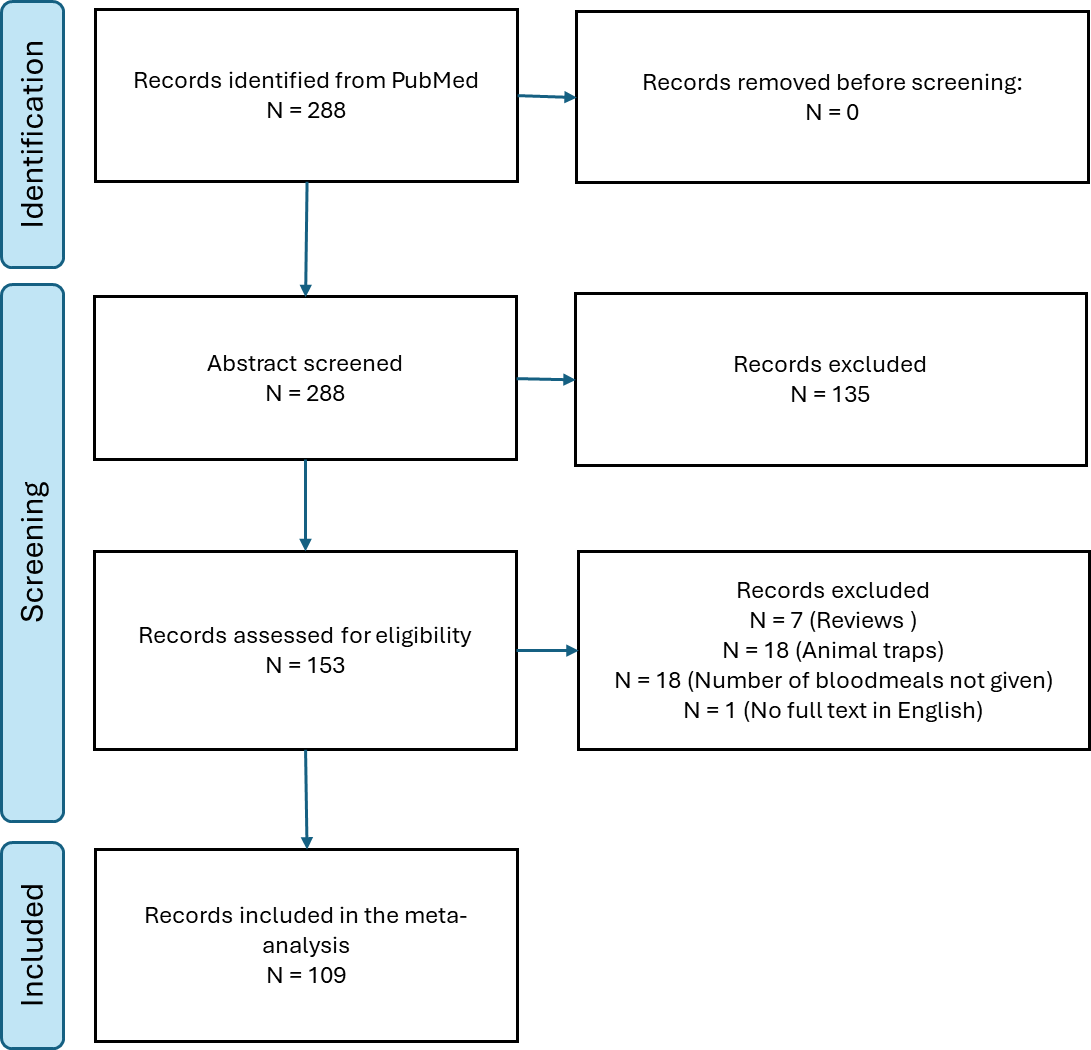
*

S1 Fig. PRISMA flowchart of selection and inclusion process for the meta-analysis of Culex blood-meal studies from the last 15 years. A total of 288 records were screened from PubMed. After the screening process 109 records were included in the meta-analysis.
