# Supplementary material for: Meta-analyses of Culex blood-meals indicates strong regional effect on feeding patterns
Source: PLoS Negl Trop Dis. 2025 Jan 24;19(1):e0012245. doi: 10.1371/journal.pntd.0012245 (PMC11785302; doi:10.1371/journal.pntd.0012245)
Supplement: S2 Fig — Culex feeding patterns across different land use types. Each figure shows the percentage of blood-meals taken on five major host groups: amphibian, avian, human, non-human mammal, reptile per mosquito species. (DOCX) [file pntd.0012245.s004.docx]

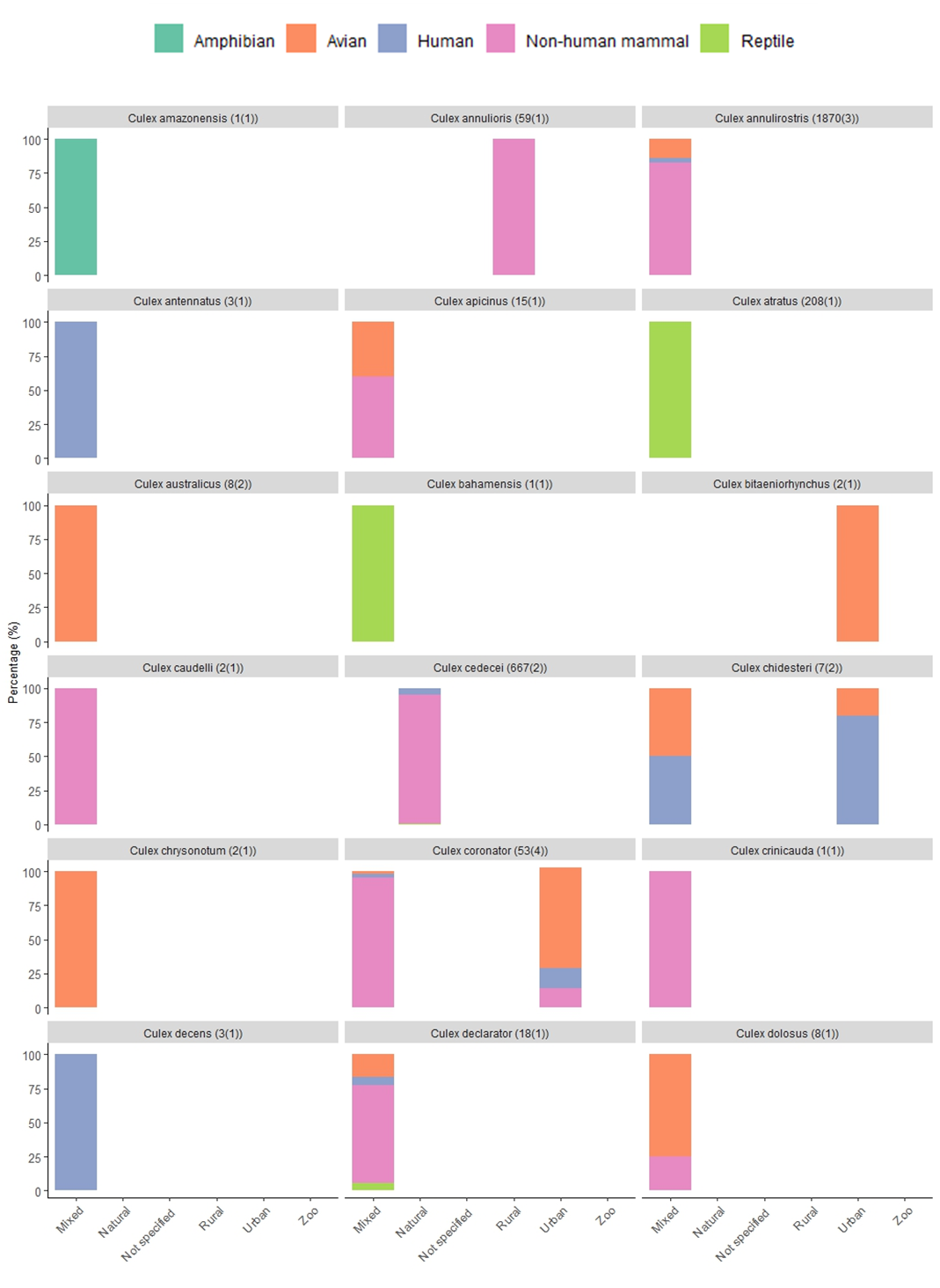


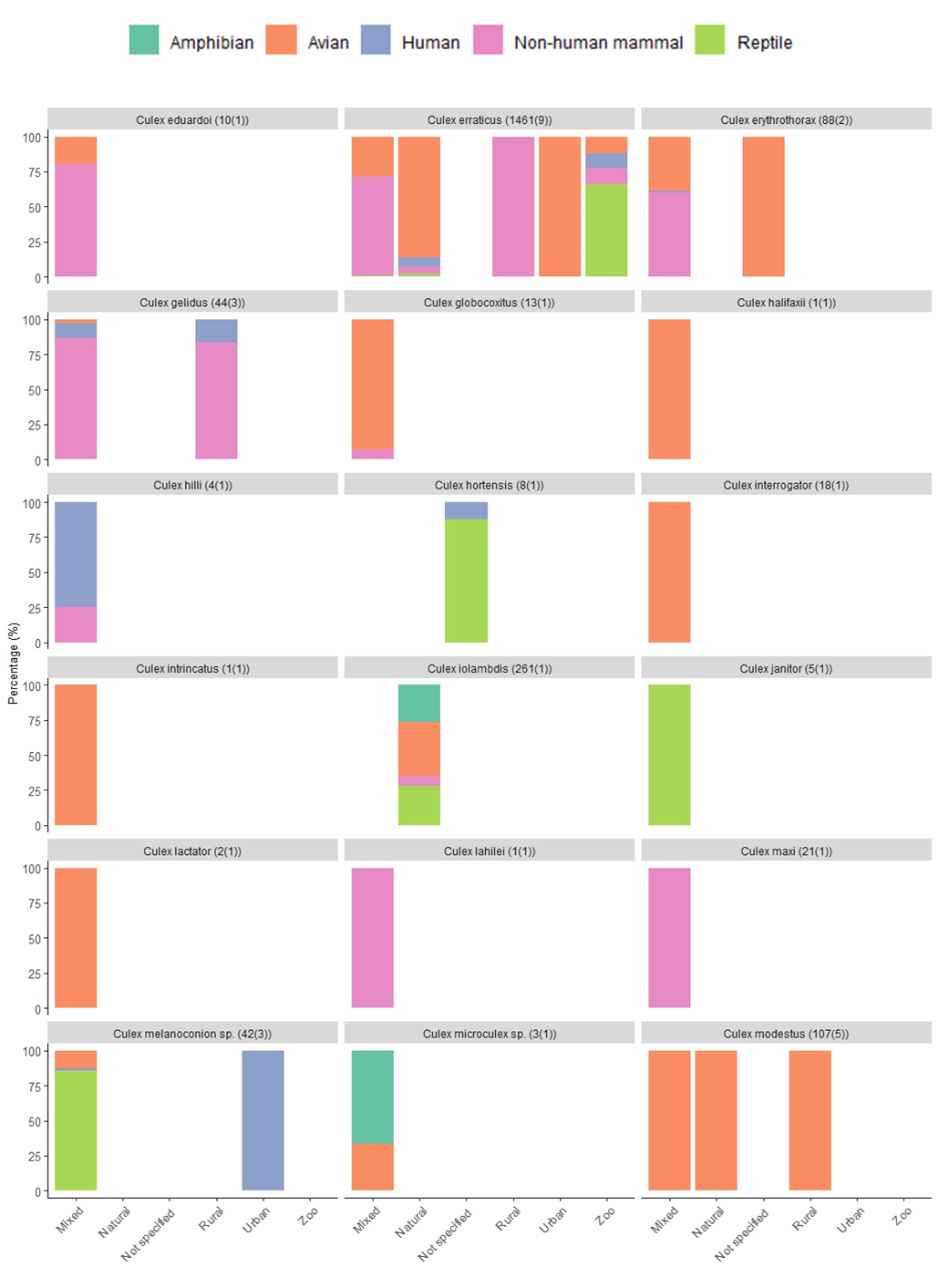


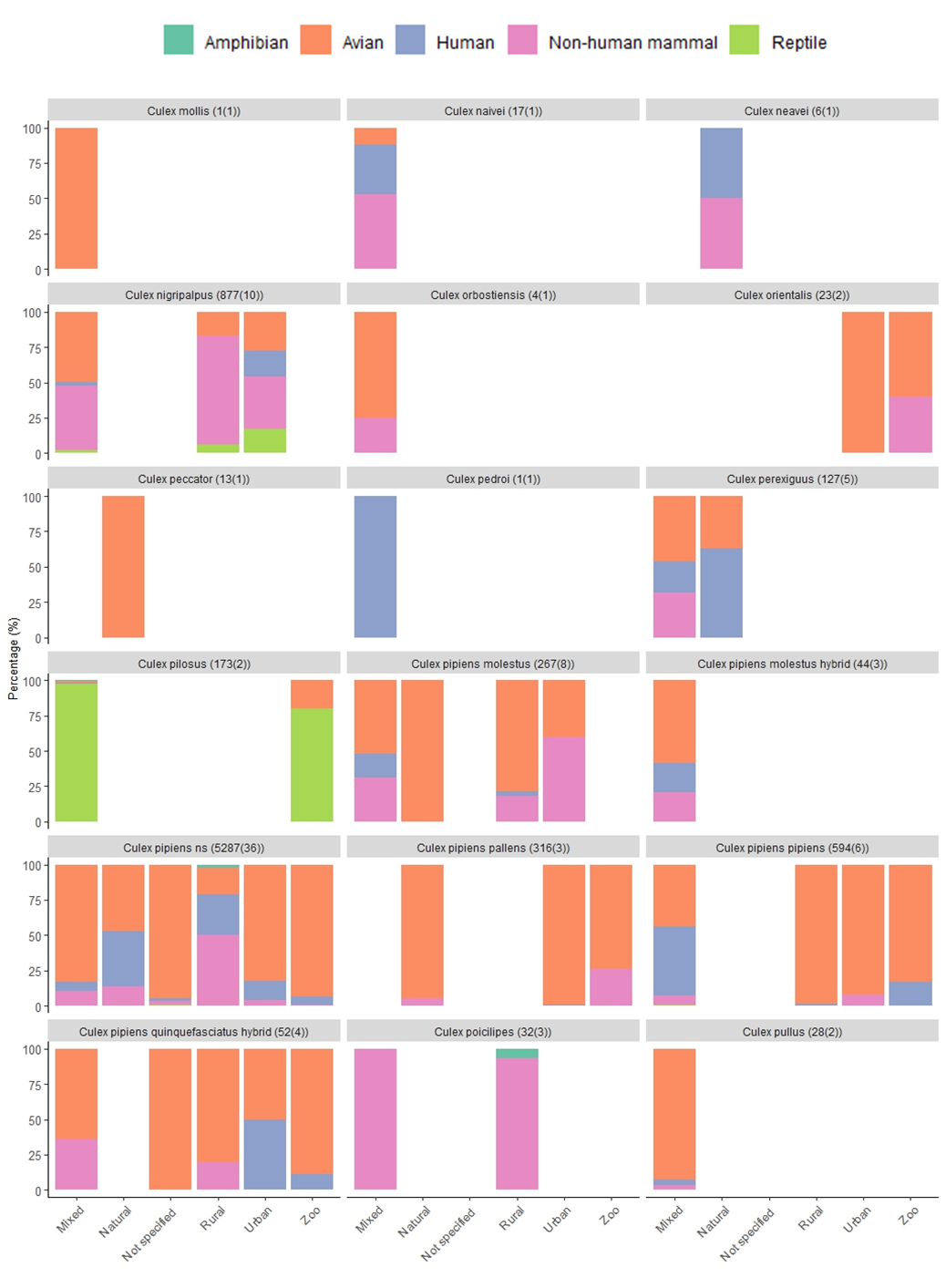


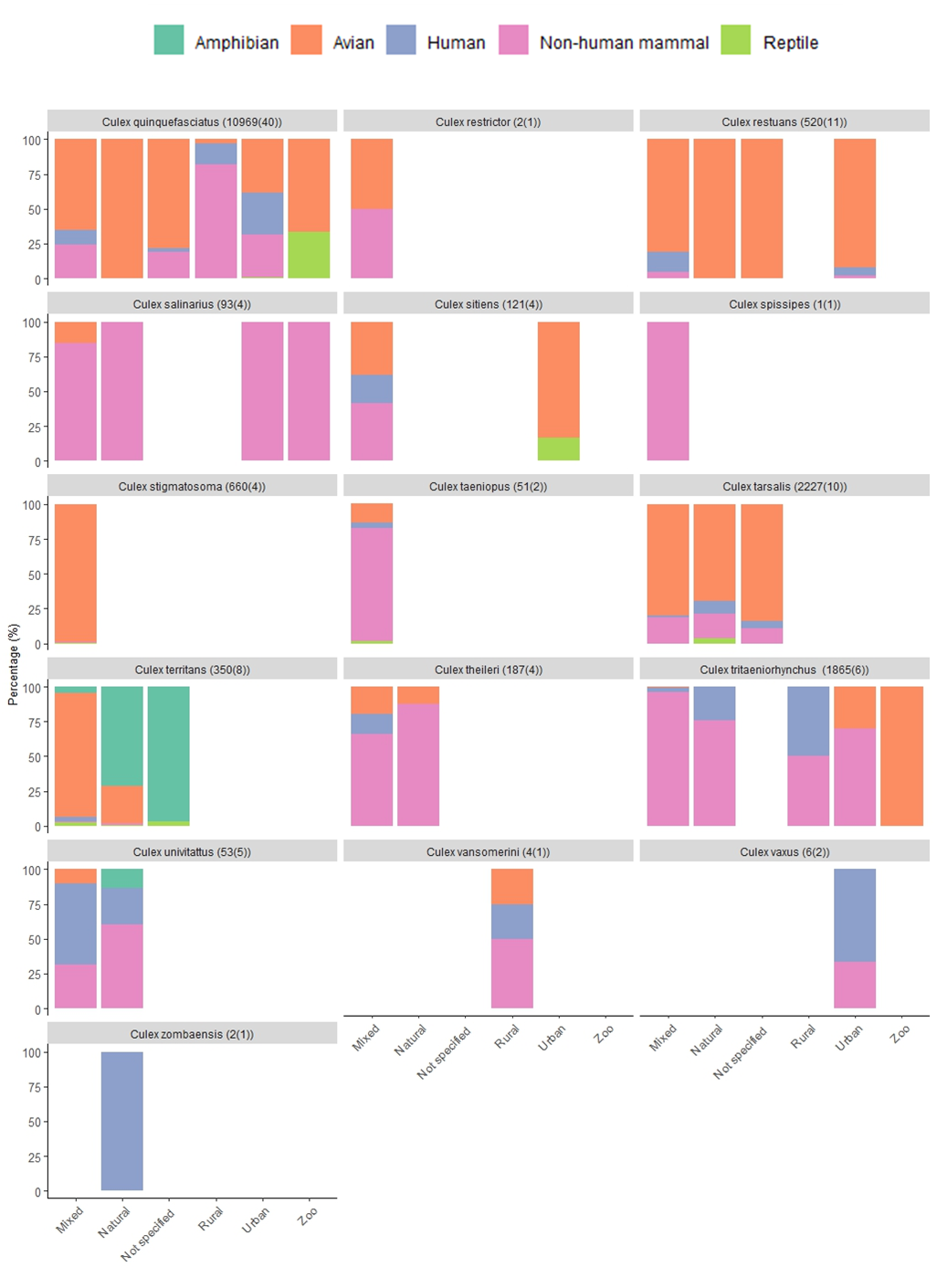


S2 Fig. *Culex* feeding patterns across different land use types. Each figure shows the percentage of blood-meals taken on five major host groups: amphibian, avian, human, non-human mammal, reptile per mosquito species. Where data was available land use from different studies was classified into six different types: mixed (mosquitoes from multiple types of land use were collected in this study), natural (land that is not modified for human use, such as wetlands, forests, grasslands, etc.), not specified (if no detailed description was given of land use the study was classified as not specified), rural (if this was described in the original publication, or the publication described low population density, small settlements, farming, and agriculture), urban (if this was described in the original publication, or the publication described a major city), and zoo. The title of each graph shows the mosquito species (Number of blood-meals (number of studies)).
