# Supplementary material for: Meta-analyses of Culex blood-meals indicates strong regional effect on feeding patterns
Source: PLoS Negl Trop Dis. 2025 Jan 24;19(1):e0012245. doi: 10.1371/journal.pntd.0012245 (PMC11785302; doi:10.1371/journal.pntd.0012245)
Supplement: S3 Fig — Percentage of blood-meals per major host group: amphibian, avian, human, non-human mammal, and reptile, that were collected using different methods, or combinations of methods. (DOCX) [file pntd.0012245.s005.docx]

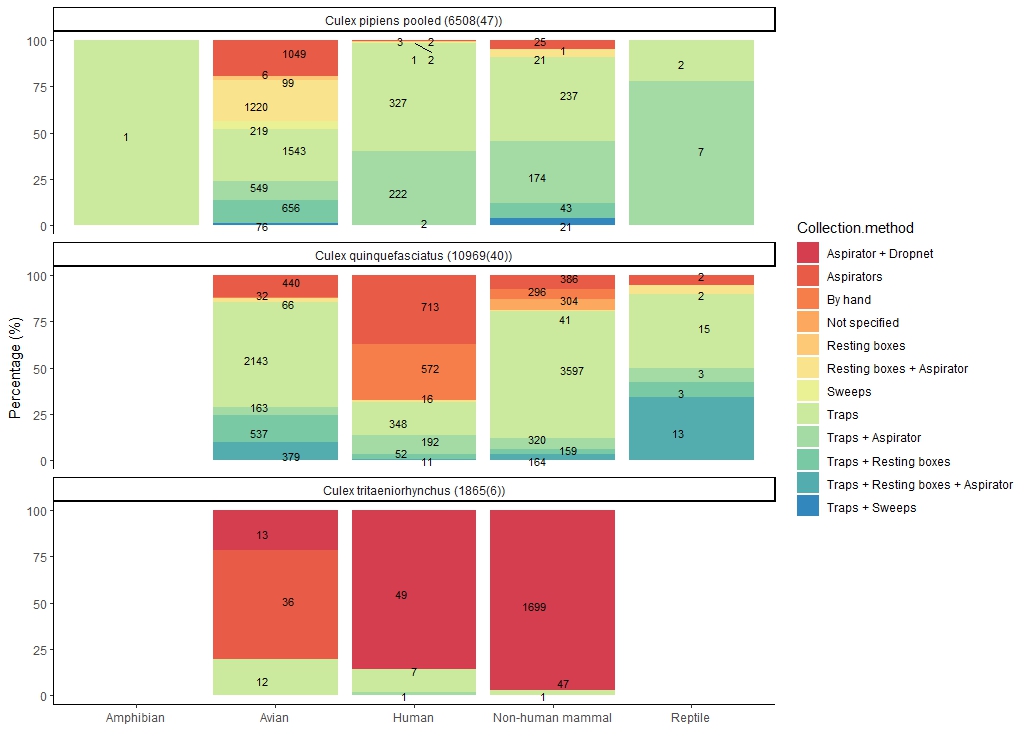


S3 Fig. Percentage of blood-meals per major host group: amphibian, avian, human, non-human mammal, and reptile, that were collected using different methods, or combinations of methods. This analysis was done for ‘Culex pipiens pooled’, Culex quinquefasciatus, Culex tritaeniorhynchus. ‘Culex pipiens pooled’ describes a pool of species that contains: Culex pipiens not specified (ns), Culex pipiens molestus, Culex pipiens pallens, Culex pipiens pipiens, Culex pipiens/molestus hybrid. The title of each graph shows the mosquito species (Number of blood-meals (number of studies)). In each bar the exact number of blood-meals collected using the different methods is shown.
