# Supplementary material for: Meta-analyses of Culex blood-meals indicates strong regional effect on feeding patterns
Source: PLoS Negl Trop Dis. 2025 Jan 24;19(1):e0012245. doi: 10.1371/journal.pntd.0012245 (PMC11785302; doi:10.1371/journal.pntd.0012245)
Supplement: S4 Fig — Percentage of blood-meals collected indoor, mixed (=indoor and outdoor), not specified, and outdoor per major host group: amphibian, avian, human, non-human mammal, and reptile. (DOCX) [file pntd.0012245.s006.docx]

*
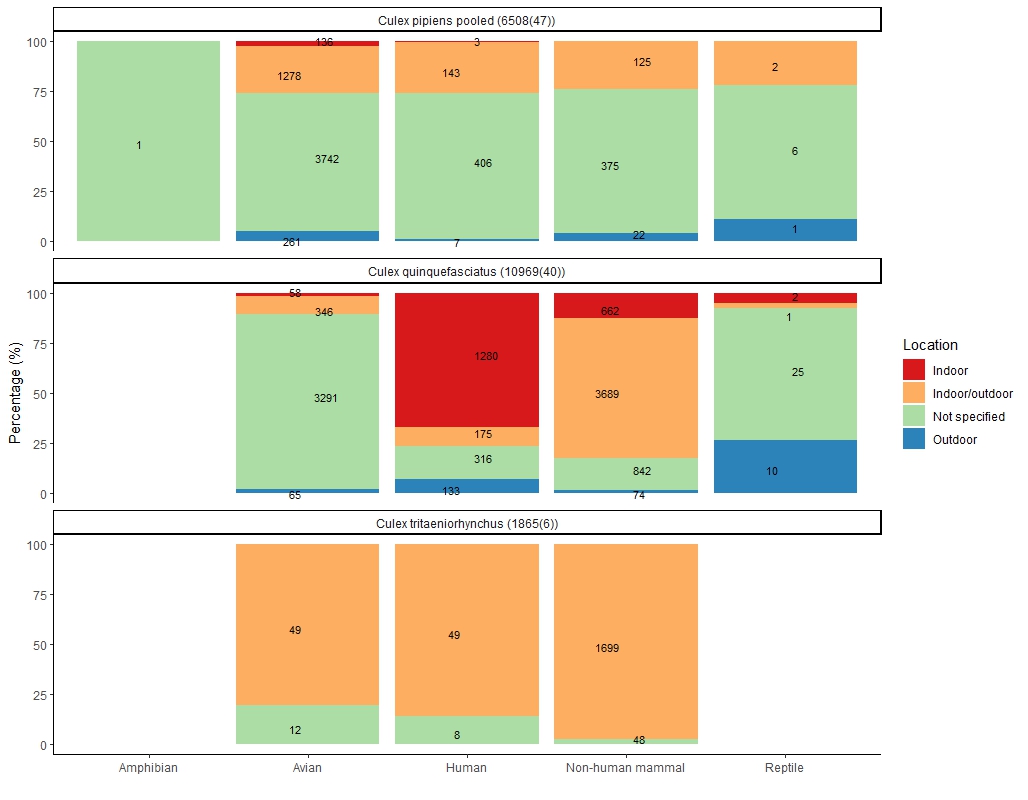
*

S4 Fig. Percentage of blood-meals collected indoor, mixed (=indoor and outdoor), not specified, and outdoor per major host group: amphibian, avian, human, non-human mammal, and reptile. This analysis was done for ‘Culex pipiens pooled’, Culex quinquefasciatus, Culex tritaeniorhynchus. ‘Culex pipiens pooled’ describes a pool of species that contains: Culex pipiens not specified (ns), Culex pipiens molestus, Culex pipiens pallens, Culex pipiens pipiens, Culex pipiens/molestus hybrid. The title of each graph shows the mosquito species (Number of blood-meals (number of studies)). In each bar the number of blood-meals is shown for each collection location.
