# Supplementary material for: Meta-analyses of Culex blood-meals indicates strong regional effect on feeding patterns
Source: PLoS Negl Trop Dis. 2025 Jan 24;19(1):e0012245. doi: 10.1371/journal.pntd.0012245 (PMC11785302; doi:10.1371/journal.pntd.0012245)
Supplement: S5 Fig — (DOCX) [file pntd.0012245.s007.docx]

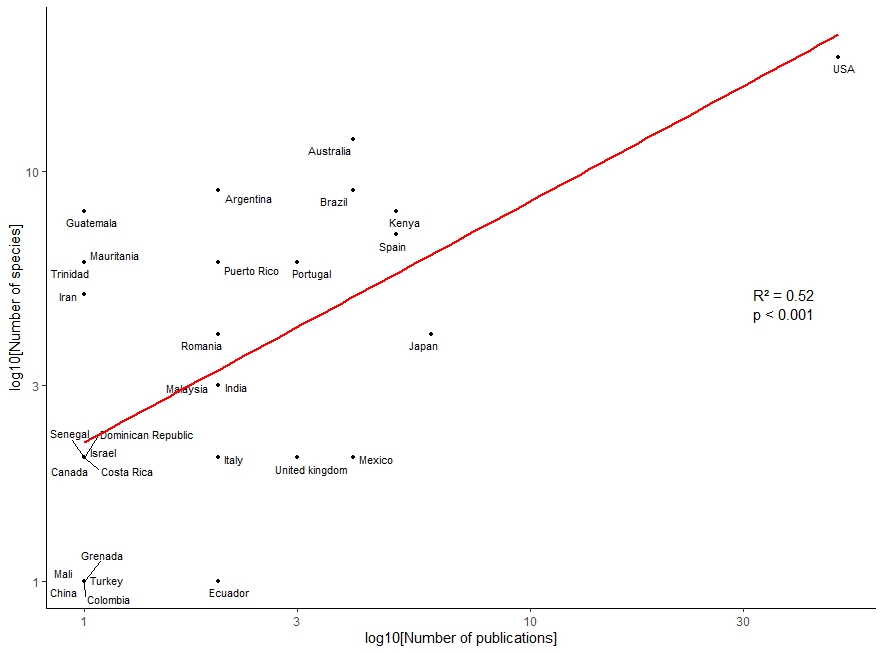


S5 Fig. Correlation between the number of publications against the number of Culex species. The circles represent the countries included in this analysis. The R-squared and p value are shown on the right side of the figure.
