# Supplementary material for: Meta-analyses of Culex blood-meals indicates strong regional effect on feeding patterns
Source: PLoS Negl Trop Dis. 2025 Jan 24;19(1):e0012245. doi: 10.1371/journal.pntd.0012245 (PMC11785302; doi:10.1371/journal.pntd.0012245)
Supplement: S6 Fig — Number of blood-meals taken from each major host group (amphibian, avian, human, non-human mammal, and reptile) for each mosquito species included in this meta-analysis. (DOCX) [file pntd.0012245.s008.docx]

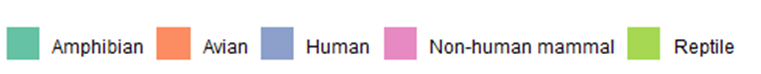

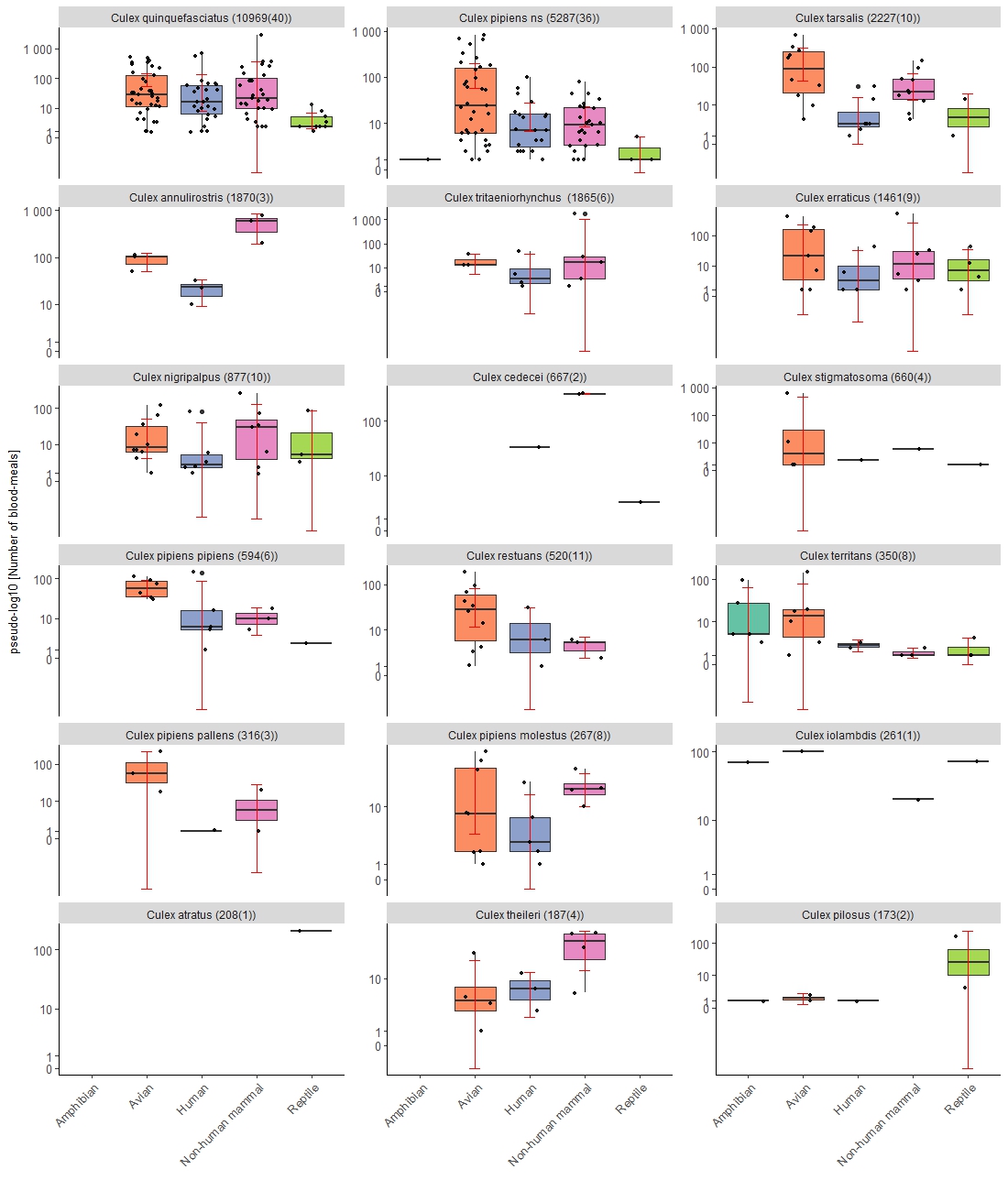


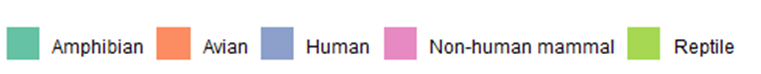


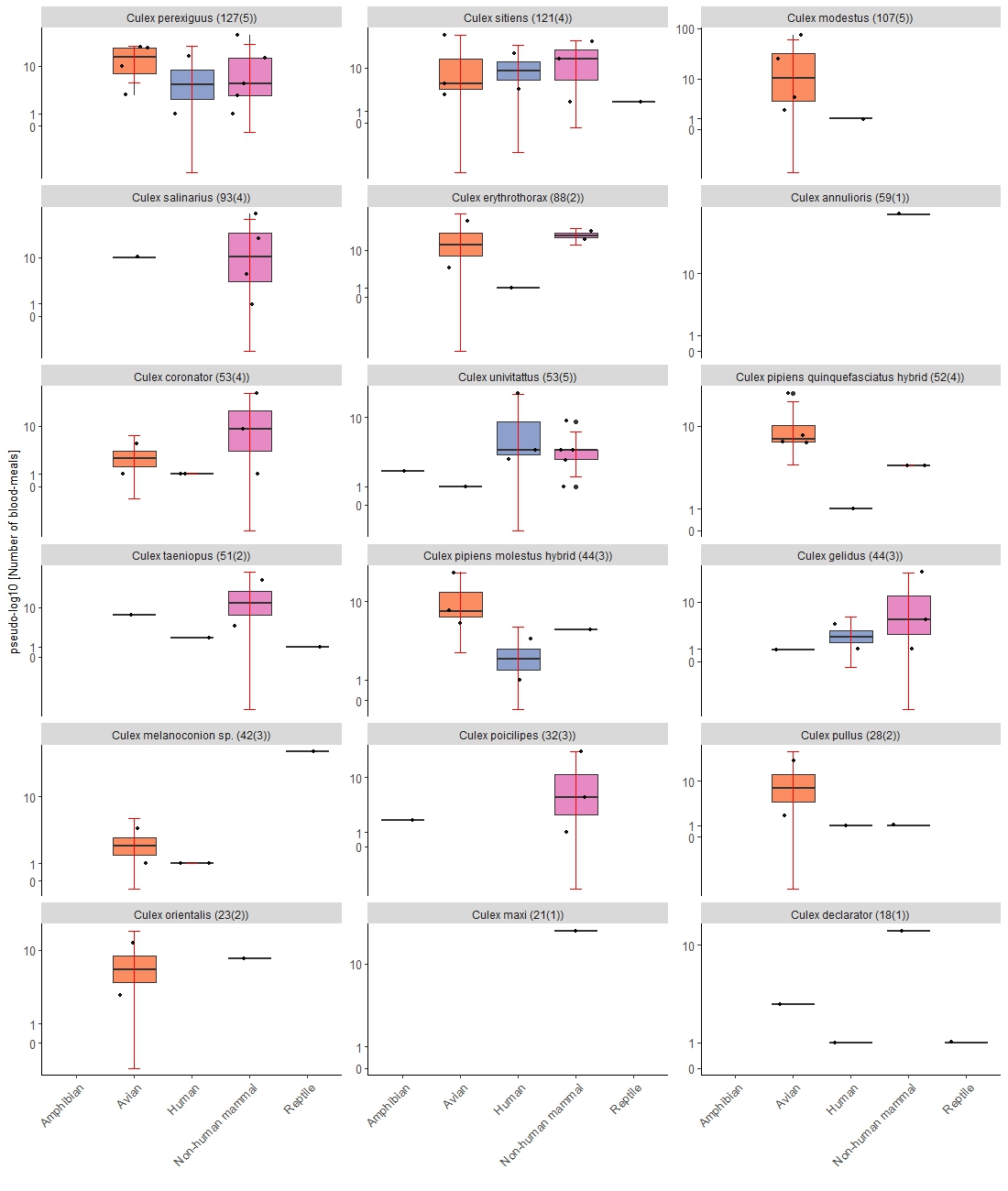


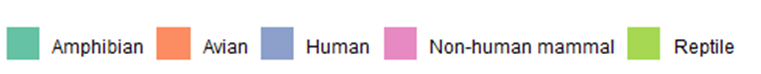

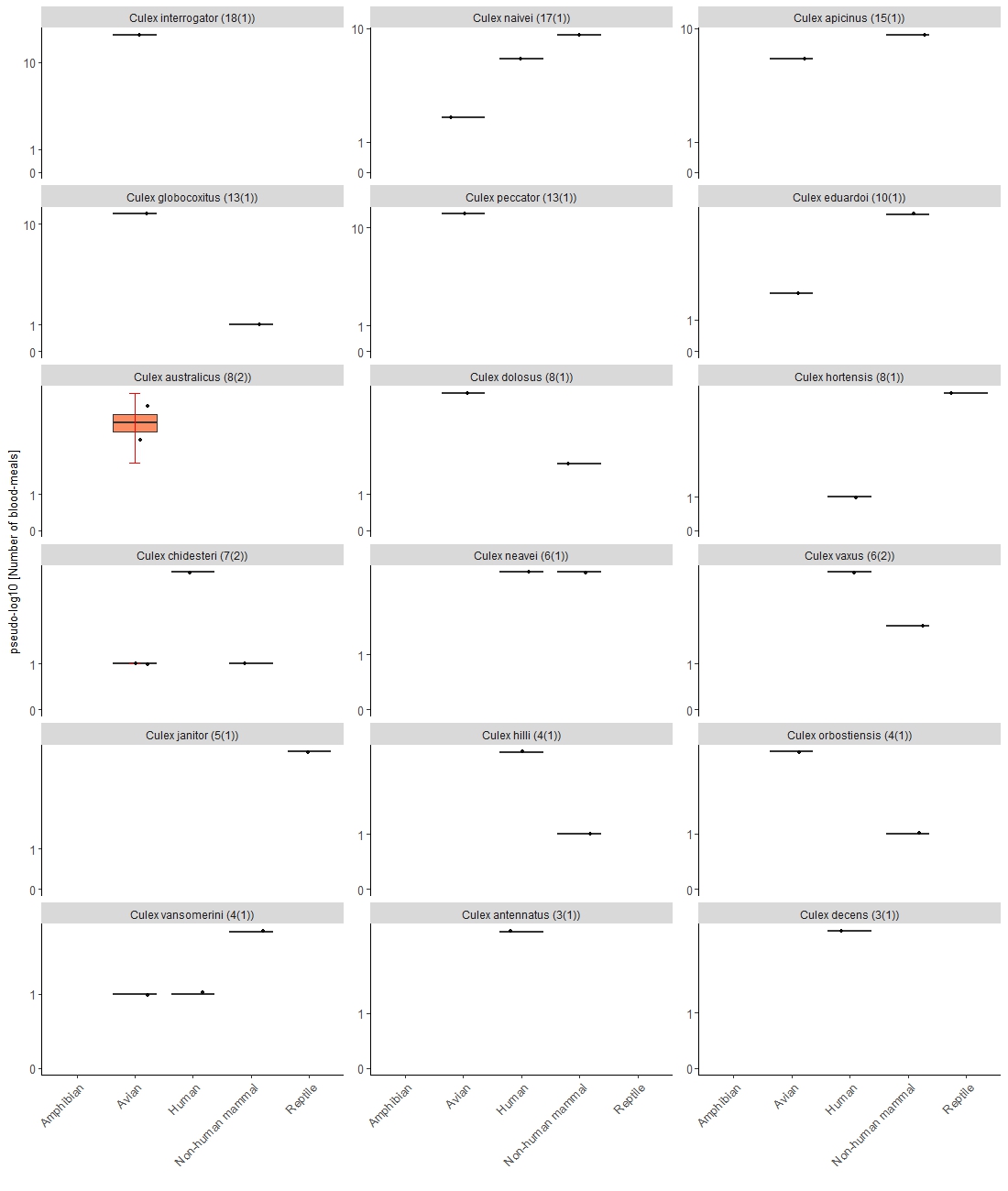


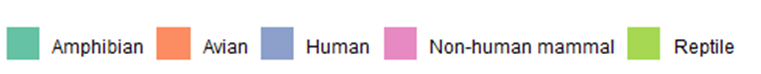

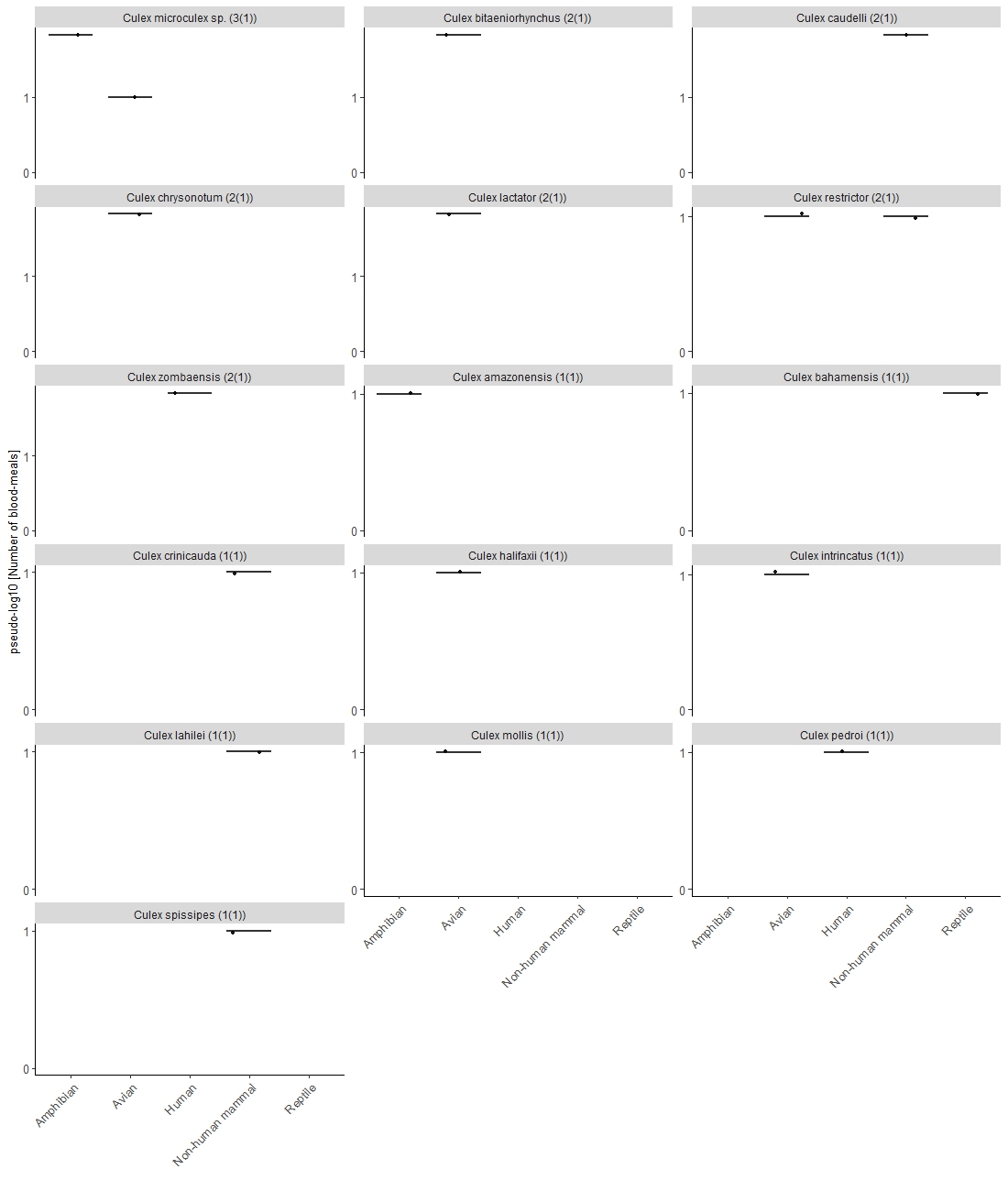


S6 Fig. Number of blood-meals taken from each major host group (amphibian, avian, human, non-human mammal, and reptile) for each mosquito species included in this meta-analysis. The title of each graph shows the mosquito species (Number of blood-meals (number of studies)). The red error bars indicate the 95% Confidence interval.
