# Supplementary material for: Meta-analyses of Culex blood-meals indicates strong regional effect on feeding patterns
Source: PLoS Negl Trop Dis. 2025 Jan 24;19(1):e0012245. doi: 10.1371/journal.pntd.0012245 (PMC11785302; doi:10.1371/journal.pntd.0012245)
Supplement: S7 Fig — Culex phylogeny and the percentage of feeding patterns on 5 major host groups (amphibian, avian, human, non-human mammal, reptile) per Culex species. The phylogenetic tree is based on the cytochrome c oxidase subunit I (COI) gene of 57 Culex (Cx.) mosquitoes. (DOCX) [file pntd.0012245.s009.docx]

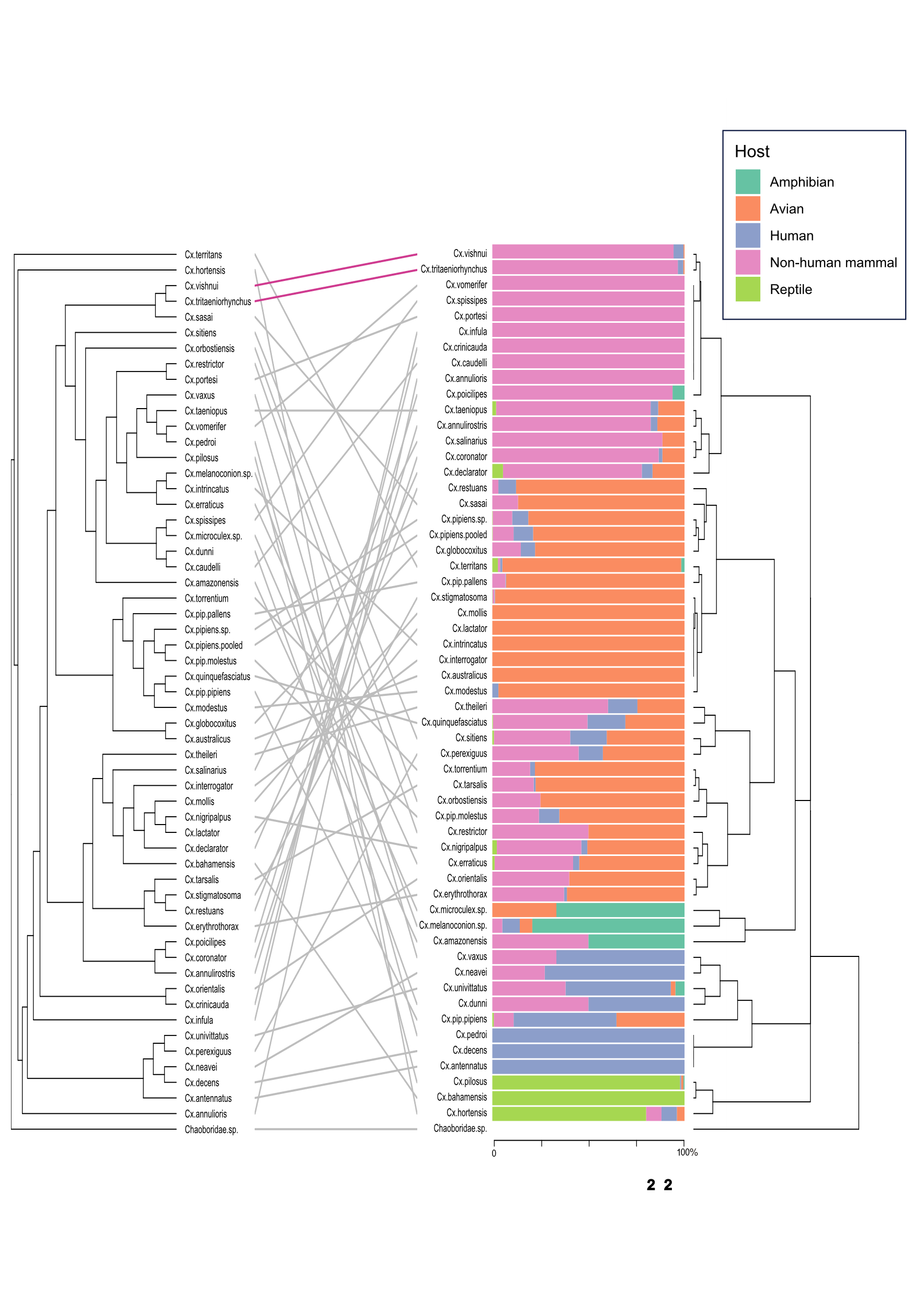
*
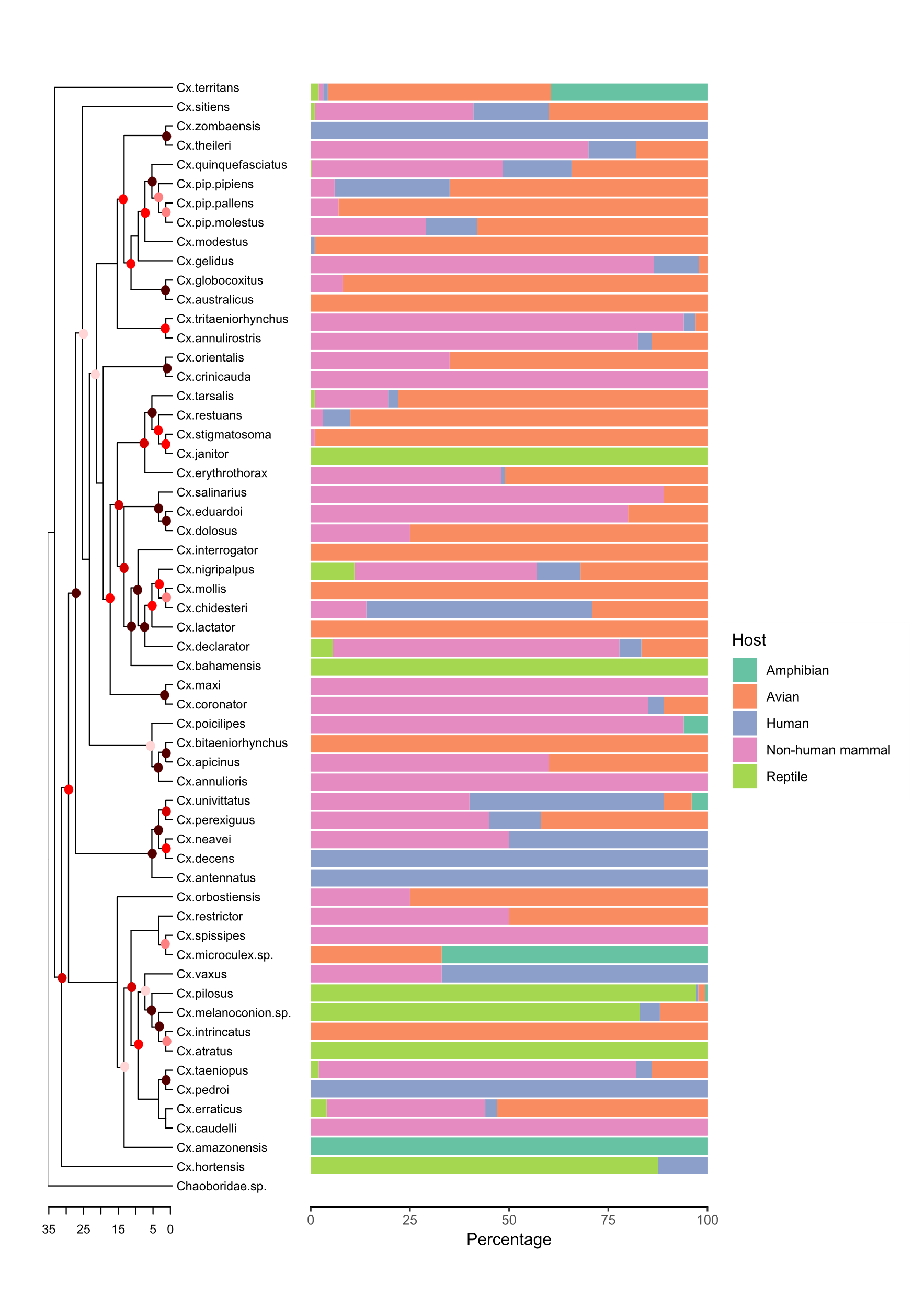
*
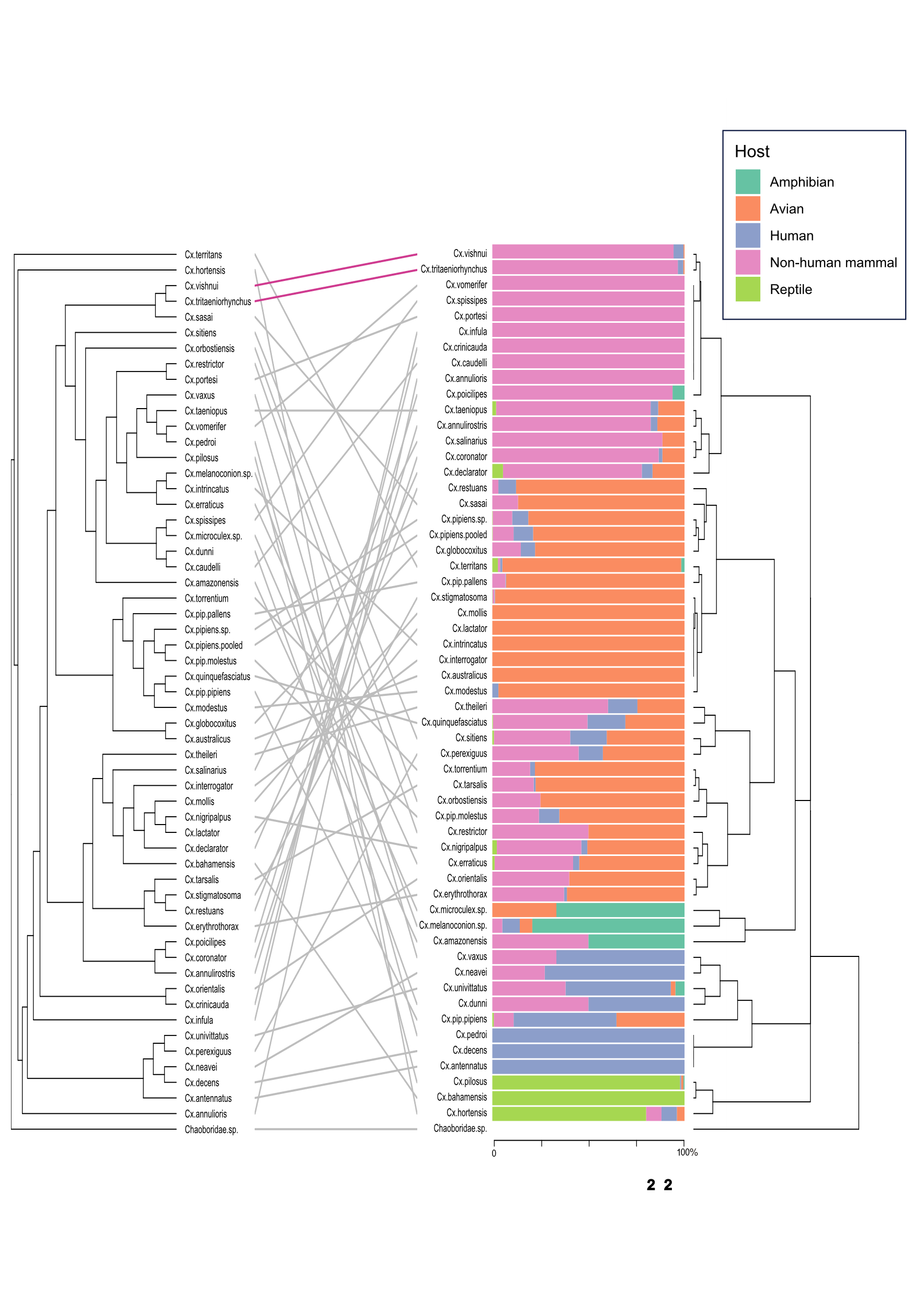


S7 Fig. Culex phylogeny and the percentage of feeding patterns on 5 major host groups (amphibian, avian, human, non-human mammal, reptile) per Culex species. The phylogenetic tree is based on the cytochrome c oxidase subunit I (COI) gene of 57 Culex (Cx.) mosquitoes. The phylogenetic tree (shown on the left) was generated using IQ-TREE with 1000 ultrafast bootstrap alignments. Node support (=bootstrap values) is indicated using coloured circles in the phylogenetic tree. If the node support was less than 50, no circle was placed on the clade.

Node support values
