# Supplementary material for: Meta-analyses of Culex blood-meals indicates strong regional effect on feeding patterns
Source: PLoS Negl Trop Dis. 2025 Jan 24;19(1):e0012245. doi: 10.1371/journal.pntd.0012245 (PMC11785302; doi:10.1371/journal.pntd.0012245)
Supplement: S8 Fig — Feeding patterns of Culex tritaeniorhynchus (A), ‘Culex pipiens pooled’ (B), and Culex quinquefasciatus across different realms. For each realm the total number of blood-meals and total number of publications (blood-meals(publications)) is shown. (DOCX) [file pntd.0012245.s010.docx]

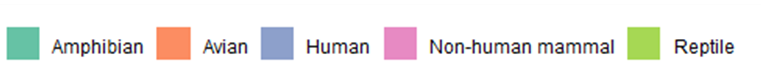


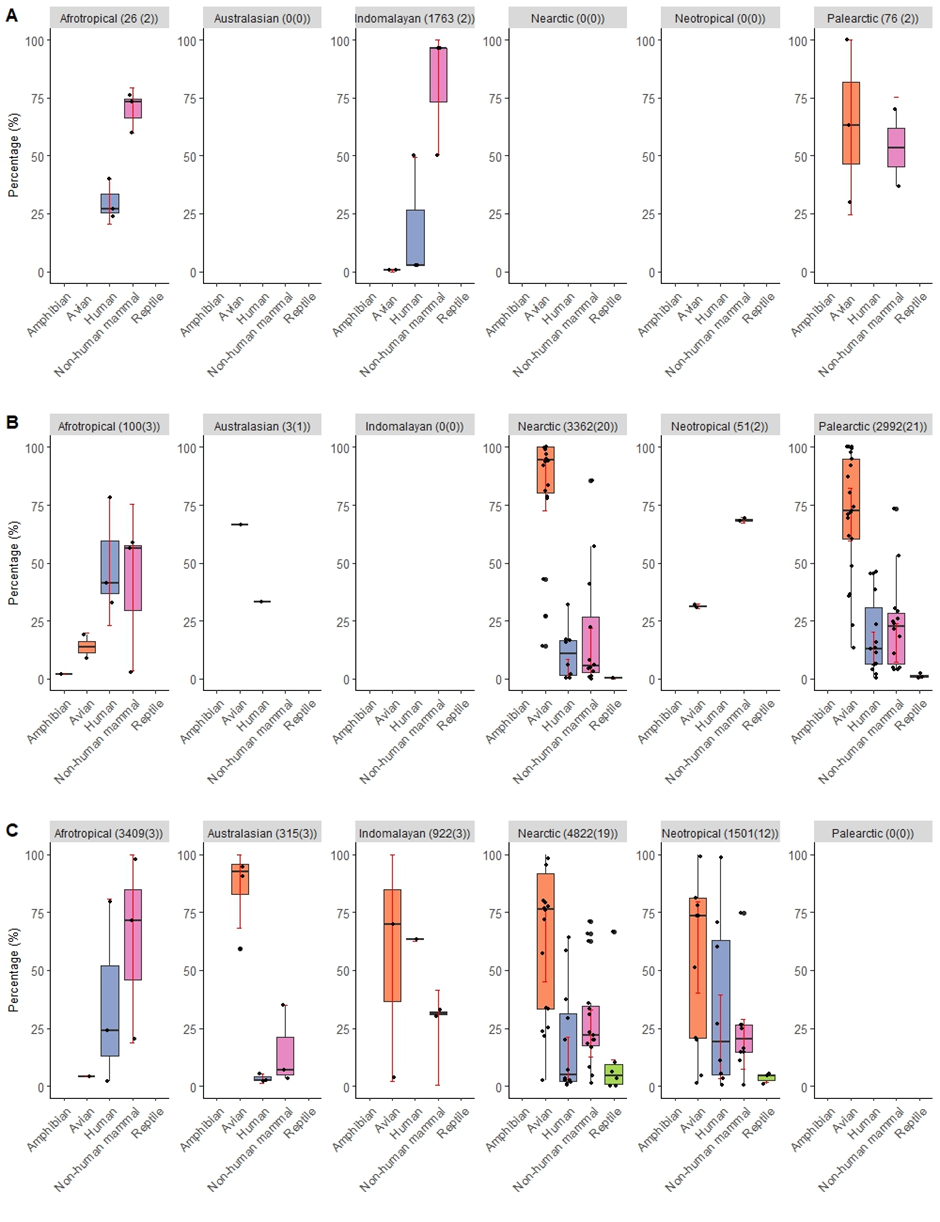


S8 Fig. Feeding patterns of Culex tritaeniorhynchus (A), ‘Culex pipiens pooled’ (B), and Culex quinquefasciatus across different realms. For each realm the total number of blood-meals and total number of publications (blood-meals(publications)) is shown. The red error bars show the 95% Confidence interval. Each dot represents a study.
