# Supplementary material for: Meta-analyses of Culex blood-meals indicates strong regional effect on feeding patterns
Source: PLoS Negl Trop Dis. 2025 Jan 24;19(1):e0012245. doi: 10.1371/journal.pntd.0012245 (PMC11785302; doi:10.1371/journal.pntd.0012245)
Supplement: S1 Table — Number of blood-meals and number of publications recorded per Culex species. For each species the references describing the blood-meals are shown. (DOCX) [file pntd.0012245.s011.docx]

S1 Table. Number of blood-meals and number of publications recorded per Culex species. For each species the references describing the bloodmeals are shown.

| **Mosquito species** | **Number of blood-meals** | **Number of publications** | **Reference** |
| --- | --- | --- | --- |
| **Culex amazonensis** | 1 | 1 | (1) |
| **Culex annulioris** | 59 | 1 | (2) |
| **Culex annulirostris** | 1870 | 3 | (3),(4),(5) |
| **Culex antennatus** | 3 | 1 | (6) |
| **Culex acinus** | 15 | 1 | (7) |
| **Culex atratus** | 208 | 1 | (8) |
| **Culex australicus** | 8 | 2 | (3),(5) |
| **Culex bahamensis** | 1 | 1 | (9) |
| **Culex bitaeniorhynchus** | 2 | 1 | (10) |
| **Culex caudelli** | 2 | 1 | (1) |
| **Culex cedecei** | 667 | 2 | (11), (12) |
| **Culex chidesteri** | 7 | 2 | (13), (7) |
| **Culex chrysonotum** | 2 | 1 | (14) |
| **Culex coronator** | 53 | 4 | (13), (15), (16), (14) |
| **Culex crinicauda** | 1 | 1 | (5) |
| **Culex decens** | 3 | 1 | (6) |
| **Culex declarator** | 18 | 1 | (1) |
| **Culex dolosus** | 8 | 1 | (7) |
| **Culex eduardoi** | 10 | 1 | (7) |
| **Culex erraticus** | 1461 | 9 | (17), (18), (15), (19), (20), (21), (22), (23), (24) |
| **Culex erythrothorax** | 88 | 2 | (25), (26) |
| **Culex gelidus** | 44 | 3 | (5), (27), (28) |
| **Culex globocoxitus** | 13 | 1 | (5) |
| **Culex halifaxii** | 1 | 1 | (3) |
| **Culex hilli** | 4 | 1 | (3) |
| **Culex hortensis** | 8 | 1 | (29) |
| **Culex interrogator** | 18 | 1 | (14) |
| **Culex intrincatus** | 1 | 1 | (30) |
| **Culex iolambdis** | 261 | 1 | (31) |
| **Culex janitor** | 5 | 1 | (9) |
| **Culex lactator** | 2 | 1 | (14) |
| **Culex lahilei** | 1 | 1 | (7) |
| **Culex melanoconion sp.** | 42 | 3 | (13), (30), (9) |
| **Culex microculex sp.** | 3 | 1 | (30) |
| **Culex modestus** | 107 | 5 | (32), (33), (34), (35), (36) |
| **Culex mollis** | 1 | 1 | (30) |
| **Culex naivei** | 17 | 1 | (37) |
| **Culex neavei** | 6 | 1 | (38) |
| **Culex nigripalpus** | 877 | 10 | (13), (39), (18), (15), (40), (16),(14), (1), (9), (41) |
| **Culex orbostiensis** | 4 | 1 | (3) |
| **Culex orientalis** | 23 | 2 | (42), (10) |
| **Culex peccator** | 13 | 1 | (17) |
| **Culex pedroi** | 1 | 1 | (1) |
| **Culex perexiguus** | 127 | 5 | (33), (34), (43), (44), (45) |
| **Culex pilosus** | 173 | 2 | (8), (21) |
| **Culex pipiens molestus** | 267 | 8 | (46), (3), (47), (48), (49), (50), (7), (51) |
| **Culex pipiens molestus hybrid** | 44 | 3 | (52), (48), (51) |
| **Culex pipiens ns** | 5287 | 36 | (53), (54), (55), (29), (56), (32), (57), (52), (58), (49), (59), (33), (60), (34), (61), (62), (63), (64), (43), (44), (65), (66), (67), (68), (36), (45), (69), (70), (71), (72), (73), (25), (74), (75), (37), (76) |
| **Culex pipiens pallens** | 316 | 3 | (47), (77), (78) |
| **Culex pipiens pipiens** | 594 | 6 | (48), (79), (50), (43), (51), (24) |
| **Culex pipiens quinquefasciatus hybrid** | 52 | 4 | (46), (52), (7), (24) |
| **Culex poicilipes** | 32 | 3 | (2), (6), (37) |
| **Culex pullus** | 28 | 2 | (3), (5) |
| **Culex quinquefasciatus** | 10969 | 40 | (17), (80), (46), (81), (13), (3), (2), (82), (52), (83), (39), (5), (84), (15), (85), (40), (86), (16), (14), (87), (7), (88), (89), (28), (90), (21), (91), (30), (9), (6), (92), (93), (41), (94), (95), (96), (24), (26), (97), (98) |
| **Culex restrictor** | 2 | 1 | (20) |
| **Culex restuans** | 520 | 11 | (17), (55), (99), (52), (79), (58), (100), (18), (63), (64), (72) |
| **Culex salinarius** | 93 | 4 | (16), (72), (73), (24) |
| **Culex sitiens** | 121 | 4 | (3), (5), (43), (90) |
| **Culex spissipes** | 1 | 1 | (1) |
| **Culex stigmatosoma** | 660 | 4 | (14), (101), (70), (26) |
| **Culex taeniopus** | 51 | 2 | (14), (9) |
| **Culex tarsalis** | 2227 | 10 | (60), (102), (103), (69), (70), (104), (105), (25), (74), (26) |
| **Culex territans** | 350 | 8 | (17), (18), (62), (63), (106), (68), (107), (108) |
| **Culex theileri** | 187 | 4 | (33), (34), (43), (44) |
| **Culex tritaeniorhynchus** | 1865 | 6 | (10), (38), (27), (28), (6), (76) |
| **Culex univitattus** | 53 | 4 | (53), (6), (94), (37) |
| **Culex vansomerini** | 4 | 1 | (37) |
| **Culex vaxus** | 6 | 1 | (109) |
| **Culex zombaensis** | 2 | 1 | (54) |

**References**

1. Kang DS, Martinez R, Hosein A, Shui Feng R, James L, Lovin DD, et al. Identification of Host Blood Meals of Mosquitoes (Diptera: Culicidae) Collected at the Aripo Savannas Scientific Reserve in Trinidad, West Indies. J Med Entomol. 2019;56(6):1734–8.

2. Muturi EJ, Muriu S, Shililu J, Mwangangi JM, Jacob BG, Mbogo C, et al. Blood-feeding patterns of Culex quinquefasciatus and other culicines and implications for disease transmission in Mwea rice scheme, Kenya. Parasitol Res. 2008;102(6):1329–35.

3. Jansen CC, Webb CE, Graham GC, Craig SB, Zborowski P, Ritchie SA, et al. Blood sources of mosquitoes collected from urban and peri-urban environments in eastern Australia with species-specific molecular analysis of avian blood meals. Am J Trop Med Hyg. 2009;81(5):849–57.

4. Hall-Mendelin S, Jansen CC, Cheah WY, Montgomery BL, Hall RA, Ritchie SA, et al. Culex annulirostris (Diptera: Culicidae) host feeding patterns and Japanese encephalitis virus ecology in Northern Australia. J Med Entomol. 2012;49(2):371–7.

5. Johansen CA, Power SL, Broom AK. Determination of mosquito (Diptera: Culicidae) bloodmeal sources in western Australia: implications for arbovirus transmission. J Med Entomol. 2009;46(5):1167–75.

6. Stoek F, Barry Y, Ba A, Schulz A, Rissmann M, Wylezich C, et al. Mosquito survey in Mauritania: Detection of Rift Valley fever virus and dengue virus and the determination of feeding patterns. PLoS Negl Trop Dis [Internet]. 2022;16(4):1–20. Available from: http://dx.doi.org/10.1371/journal.pntd.0010203

7. Cardo M V., Vezzani D. Host–mosquito interactions in rural and urban equestrian facilities from temperate Argentina. Med Vet Entomol. 2023;37(4):816–25.

8. Reeves LE, Hoyer I, Acevedo C, Burkett-Cadena ND. Host associations of Culex (Melanoconion) atratus (diptera: Culicidae) and culex (Melanoconion) pilosus from Florida, USA. Insects. 2019;10(8).

9. Barrera R, Amador M, Young G, Komar N. Mosquito (Diptera: Culicidae) bloodmeal sources during a period of west nile virus transmission in Puerto Rico. J Med Entomol. 2011;48(3):701–4.

10. Faizah AN, Kobayashi D, Matsumura R, Watanabe M, Higa Y, Sawabe K, et al. Blood meal source identification and RNA virome determination in Japanese encephalitis virus vectors collected in Ishikawa Prefecture, Japan, show distinct avian/mammalian host preference. J Med Entomol. 2023;60(3):620–8.

11. Hoyer IJ, Blosser EM, Acevedo C, Thompson AC, Reeves LE, Burkett-Cadena ND. Mammal decline, linked to invasive Burmese python, shifts host use of vector mosquito towards reservoir hosts of a zoonotic disease. Biol Lett. 2017;13(10):3–6.

12. Hoyer IJ, Acevedo C, Wiggins K, Alto BW, Burkett-Cadena ND. Patterns of abundance, host use, and everglades virus infection in Culex (Melanoconion) cedecei Mosquitoes, Florida, USA. Emerg Infect Dis. 2019;25(6):1093–100.

13. de Carvalho GC, dos Santos Malafronte R, Miti Izumisawa C, Souza Teixeira R, Natal L, Marrelli MT. Blood meal sources of mosquitoes captured in municipal parks in São Paulo, Brazil. J Vector Ecol. 2014;39(1):146–52.

14. Kading RC, Reiche ASG, Morales-Betoulle ME, Komar N. Host selection of potential West Nile virus vectors in Puerto Barrios, Guatemala, 2007. Am J Trop Med Hyg. 2013;88(1):108–15.

15. Mann JG, Washington M, Guynup T, Tarrand C, Dewey EM, Fredregill C, et al. Feeding habits of vector mosquitoes in Harris County, TX, 2018. J Med Entomol. 2020;57(6):1920–9.

16. MacKay AJ, Kramer WL, Meece JK, Brumfield RT, Foil LD. Host feeding patterns of culex mosquitoes (Diptera: Culicidae) in east baton rouge Parish, Louisiana. J Med Entomol. 2010;47(2):238–48.

17. Estep LK, McClure CJW, Burkett-Cadena ND, Hassan HK, Hicks TL, Unnasch TR, et al. A multi-year study of mosquito feeding patterns on avian hosts in a Southeastern focus of Eastern equine encephalitis virus. Am J Trop Med Hyg. 2011;84(5):718–26.

18. Burkett-Cadena ND, Bingham AM, Hunt B, Morse G, Unnasch TR. Ecology of Culiseta Melanura and Other Mosquitoes (Diptera: Culicidae) from Walton County, FL, during Winter Period 2013-2014. J Med Entomol. 2015;52(5):1074–82.

19. Mendenhall IH, Tello SA, Neira LA, Castillo LF, Ocampo CB, Wesson DM. Host preference of the arbovirus vector culex erraticus (Diptera: Culicidae) at Sonso Lake, Cauca Valley Department, Colombia. J Med Entomol. 2012;49(5):1092–102.

20. Gilkey PL, Ortiz DL, Kowalo T, Troyo A, Sirot LK. Host-feeding patterns of the mosquito assemblage at lomas barbudal biological reserve, guanacaste, costa rica. J Med Entomol. 2021;58(6):2058–66.

21. Reeves LE, Krysko KL, Avery ML, Gillett-Kaufman JL, Kawahara AY, Roxanne Connelly C, et al. Interactions between the invasive Burmese python, Python bivittatus Kuhl, and the local mosquito community in Florida, USA. PLoS One [Internet]. 2018;13(1):1–16. Available from: http://dx.doi.org/10.1371/journal.pone.0190633

22. Ber JL, Goddard J, Outlaw D. Survey of mississippi mosquito blood meals for vertebrate host identification. J Am Mosq Control Assoc. 2021;37(4):283–5.

23. Oliveira A, Katholi CR, Nathan B-C, Hassan HK, Kristensen S, Unnasch TR. Temporal analysis of feeding patterns of Culex erraticus in central Alabama. Vector-Borne Zoonotic Dis. 2011;11(4).

24. Briggs C, Osman R, Newman BC, Fikrig K, Danziger PR, Mader EM, et al. Utilization of a zoo for mosquito (Diptera: Culicidae) diversity analysis, arboviral surveillance, and blood feeding patterns. J Med Entomol. 2023;60(6):1406–17.

25. Batson J, Dudas G, Haas-Stapleton E, Kistler AL, Li LM, Logan P, et al. Single mosquito metatranscriptomics identifies vectors, emerging pathogens and reservoirs in one assay. Elife. 2021;10:1–32.

26. Molaei G, Cummings RF, Su T, Armstrong PM, Williams GA, Cheng ML, et al. Vector-host interactions governing epidemiology of West Nile virus in southern California. Am J Trop Med Hyg. 2010;83(6):1269–82.

27. Samuel PP, Arunachalam N, Hiriyan J, Tyagi BK. Host feeding pattern of Japanese encephalitis virus vector mosquitoes (Diptera: Culicidae) from Kuttanadu, Kerala, India. J Med Entomol. 2008;45(5):927–32.

28. Young KI, Medwid JT, Azar SR, Huff RM, Drumm H, Coffey LL, et al. Identification of mosquito bloodmeals collected in diverse habitats in Malaysian borneo using COI barcoding. Trop Med Infect Dis. 2020;5(2).

29. De La Puente JM, Muñoz J, Capelli G, Montarsi F, Soriguer R, Arnoldi D, et al. Avian malaria parasites in the last supper: Identifying encounters between parasites and the invasive Asian mosquito tiger and native mosquito species in Italy. Malar J. 2015;14(1):1–7.

30. Santos CS, Pie MR, da Rocha TC, Navarro-Silva MA. Molecular identification of blood meals in mosquitoes (Diptera, Culicidae) in urban and forested habitats in southern Brazil. PLoS One [Internet]. 2019;14(2):1–19. Available from: http://dx.doi.org/10.1371/journal.pone.0212517

31. Blosser EM, Stenn T, Acevedo C, Burkett-Cadena ND. Host use and seasonality of Culex (Melanoconion) iolambdis (Diptera: Culicidae) from eastern Florida, USA. Acta Trop [Internet]. Elsevier B.V.; 2016;164:352–9. Available from: http://dx.doi.org/10.1016/j.actatropica.2016.10.001

32. Brugman VA, Hernández-Triana LM, England ME, Medlock JM, Mertens PPC, Logan JG, et al. Blood-feeding patterns of native mosquitoes and insights into their potential role as pathogen vectors in the Thames estuary region of the United Kingdom. Parasites and Vectors. Parasites & Vectors; 2017;10(1):1–12.

33. Martínez-De La Puente J, Ruiz S, Soriguer R, Figuerola J. Effect of blood meal digestion and DNA extraction protocol on the success of blood meal source determination in the malaria vector Anopheles atroparvus. Malar J. 2013;12(1):2–7.

34. Muñoz J, Ruiz S, Soriguer R, Alcaide M, Viana DS, Roiz D, et al. Feeding patterns of potential West Nile virus vectors in South-West Spain. PLoS One. 2012;7(6).

35. Guo XX, Zhang YM, Li CX, Zhang GL, Zheng Z, Dong Y De, et al. Host-Seeking Behavior and Arbovirus Detection in Mosquitoes of Habahe County, Xinjiang Uigur Autonomous Region, China. J Am Mosq Control Assoc. 2015;31(4):329–35.

36. Hernández-Triana LM, Brugman VA, Prosser SWJ, Weland C, Nikolova N, Thorne L, et al. Molecular approaches for blood meal analysis and species identification of mosquitoes (Insecta: Diptera: Culicidae) in rural locations in southern England, United Kingdom. Zootaxa. 2017;4250(1):67–76.

37. Omondi D, Masiga DK, Ajamma YU, Fielding BC, Njoroge L, Villinger J. Unraveling host-vector-arbovirus interactions by two-gene high resolution melting mosquito bloodmeal analysis in a Kenyan wildlife-livestock interface. PLoS One. 2015;10(7):1–13.

38. Fall AG, Diaïté A, Lancelot R, Tran A, Soti V, Etter E, et al. Feeding behaviour of potential vectors of West Nile virus in Senegal. Parasites and Vectors [Internet]. BioMed Central Ltd; 2011;4(1):99. Available from: http://www.parasitesandvectors.com/content/4/1/99

39. Unlu I, Kramer WL, Roy AF, Foil LD. Detection of west nile virus RNA in mosquitoes and identification of mosquito blood meals collected at alligator farms in Louisiana. J Med Entomol. 2010;47(4):625–33.

40. Hancock C, Camp J V. Habitat-Specific Host Selection Patterns of Culex Quinquefasciatus and Culex Nigripalpus in Florida. J Am Mosq Control Assoc. 2022;38(2):89–91.

41. González MA, Bravo-Barriga D, Rodríguez-Sosa MA, Rueda J, Frontera E, Alarcón-Elbal PM. Species Diversity, Habitat Distribution, and Blood Meal Analysis of Haematophagous Dipterans Collected by CDC-UV Light Traps in the Dominican Republic. Pathogens. 2022;11(7).

42. Ejiri H, Sato Y, Kim KS, Tsuda Y, Murata K, Saito K, et al. Blood meal identification and prevalence of Avian Malaria parasite in mosquitoes collected at Kushiro Wetland, A Subarctic Zone of Japan. J Med Entomol. 2011;48(4):904–8.

43. Shahhosseini N, Friedrich J, Moosa-Kazemi SH, Sedaghat MM, Kayedi MH, Tannich E, et al. Host-feeding patterns of Culex mosquitoes in Iran. Parasites and Vectors. Parasites & Vectors; 2018;11(1):1–10.

44. Osório HC, Zé-Zé L, Alves MJ. Host-feeding patterns of Culex pipiens and other potential mosquito vectors (Diptera: Culicidae) of West Nile Virus (Flaviviridae) collected in Portugal. J Med Entomol. 2012;49(3):717–21.

45. Valinsky L, Ettinger C, Bar-Gal GK, Orshan L. Molecular identification of bloodmeals from sand flies and mosquitoes collected in Israel. J Med Entomol. 2014;51(3):678–85.

46. Cardo MV, Carbajo AE, Mozzoni C, Kliger M, Vezzani D. Blood feeding patterns of the Culex pipiens complex in equestrian land uses and their implications for arboviral encephalitis risk in temperate Argentina. Zoonoses Public Health. 2023;70(3):256–68.

47. Kim KS, Tsuda Y, Yamada A. Bloodmeal identification and detection of avian malaria parasite from mosquitoes (Diptera: Culicidae) inhabiting coastal areas of Tokyo Bay, Japan. J Med Entomol. 2009;46(5):1230–4.

48. Tiron GV, Stancu IG, DInu S, Prioteasa FL, Falcuta E, Ceianu CS, et al. Characterization and Host-Feeding Patterns of Culex pipiens s.l. Taxa in a West Nile Virus-Endemic Area in Southeastern Romania. Vector-Borne Zoonotic Dis. 2021;21(9):713–9.

49. Martínez-De La Puente J, Ferraguti M, Ruiz S, Roiz D, Soriguer RC, Figuerola J. Culex pipiens forms and urbanization: Effects on blood feeding sources and transmission of avian Plasmodium. Malar J. BioMed Central; 2016;15(1):1–8.

50. Gomes B, Sousa CA, Vicente JL, Pinho L, Calderón I, Arez E, et al. Feeding patterns of molestus and pipiens forms of Culex pipiens (Diptera: Culicidae) in a region of high hybridization. Parasites and Vectors. 2013;6(1):1–10.

51. Osório HC, Zé-Zé L, Amaro F, Nunes A, Alves MJ. Sympatric occurrence of Culex pipiens (Diptera, Culicidae) biotypes pipiens, molestus and their hybrids in Portugal, Western Europe: Feeding patterns and habitat determinants. Med Vet Entomol. 2014;28(1):103–9.

52. Kothera L, Mutebi JP, Kenney JL, Saxton-Shaw K, Ward MP, Savage HM, et al. Bloodmeal, Host Selection, and Genetic Admixture Analyses of Culex pipiens Complex (Diptera: Culicidae) Mosquitoes in Chicago, IL. J Med Entomol. 2020;57(1):78–87.

53. Ogola EO, Bastos ADS, Rotich G, Kopp A, Slothouwer I, Omoga DCA, et al. Analyses of Mosquito Species Composition, Blood-Feeding Habits and Infection with Insect-Specific Flaviviruses in Two Arid, Pastoralist-Dominated Counties in Kenya. Pathogens. 2023;12(7).

54. Musa AA, Muturi MW, Musyoki AM, Ouso DO, Oundo JW, Makhulu EE, et al. Arboviruses and blood meal sources in zoophilic mosquitoes at human-wildlife interfaces in Kenya. Vector-Borne Zoonotic Dis. 2020;20(6):444–53.

55. Diuk-Wasser MA, Molaei G, Simpson JE, Folsom-O’Keefe CM, Armstrong PM, Andreadis TG. Avian communal roosts as amplification foci for West Nile virus in urban areas in Northeastern United States. Am J Trop Med Hyg. 2010;82(2):337–43.

56. Hernandez-Colina A, Gonzalez-Olvera M, Lomax E, Townsend F, Maddox A, Hesson JC, et al. Blood-feeding ecology of mosquitoes in two zoological gardens in the United Kingdom. Parasites and Vectors [Internet]. BioMed Central; 2021;14(1):1–18. Available from: https://doi.org/10.1186/s13071-021-04735-0

57. Montgomery MJ, Thiemann T, Macedo P, Brown DA, Scott TW. Blood-feeding patterns of the Culex pipiens complex in Sacramento and Yolo Counties, California. J Med Entomol. 2011;48(2):398–404.

58. Hamer GL, Kitron UD, Brawn JD, Loss SR, Ruiz MO, Goldberg TL, et al. Culex pipiens (Diptera: Culicidae): A bridge vector of West Nile virus to humans. J Med Entomol. 2008;45(1):125–8.

59. Tomazatos A, Jansen S, Pfister S, Török E, Maranda I, Horv C, et al. Ecology of West Nile virus in the Danube Delta, Romania: Phylogeography, Xenosurveillance and Mosquito Host-Feeding patterns. 2019;1–19.

60. Hartman DA, Rice LM, DeMaria J, Borland EM, Bergren NA, Fagre AC, et al. Entomological risk factors for potential transmission of Rift Valley fever virus around concentrations of livestock in Colorado. Transbound Emerg Dis. 2019;66(4):1709–17.

61. Khalil N, Little EAH, Akaratovic KI, Kiser JP, Abadam CF, Yuan KJ, et al. Host Associations of Culex pipiens: A Two-Year Analysis of Bloodmeal Sources and Implications for Arboviral Transmission in Southeastern Virginia. Vector-Borne Zoonotic Dis. 2021;21(12):961–72.

62. Shepard JJ, Andreadis TG, Thomas MC, Molaei G. Host associations of mosquitoes at eastern equine encephalitis virus foci in Connecticut, USA. Parasites and Vectors [Internet]. Parasites & Vectors; 2016;9(1). Available from: http://dx.doi.org/10.1186/s13071-016-1765-1

63. Cohen SB, Lewoczko K, Huddleston DB, Moody E, Mukherjee S, Dunn JR, et al. Host feeding patterns of potential vectors of eastern equine encephalitis virus at an epizootic focus in Tennessee. Am J Trop Med Hyg. 2009;81(3):452–6.

64. Hamer GL, Kitron UD, Goldberg TL, Brawn JD, Loss SR, Ruiz MO, et al. Host selection by Culex pipiens mosquitoes and west nile virus amplification. Am J Trop Med Hyg. 2009;80(2):268–78.

65. Muñoz JN, Eritja R, Alcaide M, Montalvo T, Soriguer RC, Figuerola J. Host-feeding patterns of native culex pipiens and invasive aedes albopictus mosquitoes (Diptera: Culicidae) in Urban Zones from Barcelona, Spain. J Med Entomol. 2011;48(4):956–60.

66. Boothe E, Medeiros MCI, Kitron UD, Brawn JD, Ruiz MO, Goldberg TL, et al. Identification of avian and hemoparasite DNA in blood-engorged abdomens of Culex pipiens (diptera; Culicidae) from a west Nile virus epidemic region in suburban Chicago, Illinois. J Med Entomol. 2015;52(3):461–8.

67. Molaei G, Armstrong PM, Graham AC, Kramer LD, Andreadis TG. Insights into the recent emergence and expansion of eastern equine encephalitis virus in a new focus in the Northern New England USA. Parasites and Vectors [Internet]. Parasites & Vectors; 2015;8(1):1–15. Available from: http://dx.doi.org/10.1186/s13071-015-1145-2

68. Shahhosseini N, Frederick C, Racine T, Kobinger GP, Wong G. Modeling host-feeding preference and molecular systematics of mosquitoes in different ecological niches in Canada. Acta Trop [Internet]. Elsevier B.V.; 2021;213(November 2020):105734. Available from: https://doi.org/10.1016/j.actatropica.2020.105734

69. Mehus JO, Vaughan JA. Molecular identification of vertebrate and hemoparasite DNA within mosquito blood meals from eastern North dakota. Vector-Borne Zoonotic Dis. 2013;13(11):818–24.

70. Wheeler SS, Taff CC, Reisen WK, Townsend AK. Mosquito blood-feeding patterns and nesting behavior of American crows, an amplifying host of West Nile virus. Parasites and Vectors [Internet]. BioMed Central; 2021;14(1):1–12. Available from: https://doi.org/10.1186/s13071-021-04827-x

71. Martínez-de la Puente J, Soriguer R, Senar JC, Figuerola J, Bueno-Mari R, Montalvo T. Mosquitoes in an Urban Zoo: Identification of Blood Meals, Flight Distances of Engorged Females, and Avian Malaria Infections. Front Vet Sci. 2020;7(August):10–5.

72. Goodman H, Egizi A, Fonseca DM, Leisnham PT, Ladeau SL. Primary blood-hosts of mosquitoes are influenced by social and ecological conditions in a complex urban landscape. Parasites and Vectors. Parasites & Vectors; 2018;11(1):1–10.

73. Anderson JF, Armstrong PM, Misencik MJ, Bransfield AB, Andreadis TG, Molaei G. Seasonal Distribution, Blood-Feeding Habits, and Viruses of Mosquitoes in an Open-Faced Quarry in Connecticut, 2010 and 2011. J Am Mosq Control Assoc. 2018;34(1):1–10.

74. Thiemann TC, Lemenager DA, Kluh S, Carroll BD, Lothrop HD, Reisen WK. Spatial variation in host feeding patterns of culex tarsalis and the culex pipiens complex (Diptera: Culicidae) in California. J Med Entomol. 2012;49(4):903–16.

75. Rizzoli A, Bolzoni L, Chadwick EA, Capelli G, Montarsi F, Grisenti M, et al. Understanding West Nile virus ecology in Europe: Culex pipiens host feeding preference in a hotspot of virus emergence. Parasites and Vectors [Internet]. ???; 2015;8(1):1–13. Available from: ???

76. Inumaru M, Yamada A, Shimizu M, Ono A, Horinouchi M, Shimamoto T, et al. Vector incrimination and transmission of avian malaria at an aquarium in Japan: mismatch in parasite composition between mosquitoes and penguins. Malar J [Internet]. BioMed Central; 2021;20(1):1–12. Available from: https://doi.org/10.1186/s12936-021-03669-3

77. Ejiri H, Sato Y, Kim KS, Hara T, Tsuda Y, Imura T, et al. Entomological study on transmission of avian malaria parasites in a zoological garden in Japan: Bloodmeal identification and detection of avian malaria parasite DNA from blood-fed mosquitoes. J Med Entomol. 2011;48(3):600–7.

78. Kim KS, Tsuda Y. Seasonal changes in the feeding pattern of Culex pipiens pallens govern the transmission dynamics of multiple lineages of avian malaria parasites in Japanese wild bird community. Mol Ecol. 2010;19(24):5545–54.

79. Faraji A, Egizi A, Fonseca DM, Unlu I, Crepeau T, Healy SP, et al. Comparative Host Feeding Patterns of the Asian Tiger Mosquito, Aedes albopictus, in Urban and Suburban Northeastern USA and Implications for Disease Transmission. PLoS Negl Trop Dis. 2014;8(8).

80. Asigau S, Salah S, Parker PG. Assessing the blood meal hosts of Culex quinquefasciatus and Aedes taeniorhynchus in Isla Santa Cruz, Galápagos. Parasites and Vectors [Internet]. BioMed Central; 2019;12(1):1–10. Available from: https://doi.org/10.1186/s13071-019-3835-7

81. Lorosa ES, Faria MS, De Oliveira LCM, Alencar J, Marcondes CB. Blood meal identification of selected mosquitoes in Rio de Janeiro, Brazil. J Am Mosq Control Assoc. 2010;26(1):18–23.

82. Hannon ER, Jackson KC, Biggerstaff BJ, Raman V, Komar N. Bloodmeal Host Selection of Culex quinquefasciatus (Diptera: Culicidae) in Las Vegas, Nevada, United States. J Med Entomol. 2019;56(3):603–8.

83. Guinn A, Su T, Thieme J, Cheng ML, Brown MQ, Thiemann T. Characterization of the Blood-Feeding Patterns of Culex quinquefasciatus (Diptera: Culicidae) in San Bernardino County, California. J Med Entomol. 2022;59(5):1756–65.

84. Brown R, Hing CT, Fornace K, Ferguson HM. Evaluation of resting traps to examine the behaviour and ecology of mosquito vectors in an area of rapidly changing land use in Sabah, Malaysian Borneo. Parasites and Vectors. Parasites & Vectors; 2018;11(1):1–15.

85. Komar N, Panella NA, Golnar AJ, Hamer GL. Forage Ratio Analysis of the Southern House Mosquito in College Station, Texas. Vector-Borne Zoonotic Dis. 2018;18(9):485–90.

86. Olson MF, Ndeffo-Mbah ML, Juarez JG, Garcia-Luna S, Martin E, Borucki MK, et al. High rate of non-human feeding by aedes aegypti reduces zika virus transmission in South Texas. Viruses. 2020;12(4):1–21.

87. Garcia-Rejon JE, Blitvich BJ, Farfan-Ale JA, Loroño-Pino MA, Chi Chim WA, Flores-Flores LF, et al. Host-feeding preference of the mosquito, Culex quinquefasciatus, in Yucatan State, Mexico. J Insect Sci. 2010;10(1):1–12.

88. Pruszynski CA, Stenn T, Acevedo C, Leal AL, Burkett-Cadena ND. Human blood feeding by Aedes aegypti (diptera: culicidae) in the Florida Keys and a review of the literature. J Med Entomol. 2020;57(5):1640–7.

89. Greenberg JA, Lujan DA, Dimenna MA, Wearing HJ, Hofkin B V. Identification of blood meal sources in aedes vexans and culex quinquefasciatus in Bernalillo County, New Mexico. J Insect Sci. 2013;13.

90. Gyawali N, Taylor-Robinson AW, Bradbury RS, Huggins DW, Hugo LE, Lowry K, et al. Identification of the source of blood meals in mosquitoes collected from north-eastern Australia. Parasites and Vectors [Internet]. BioMed Central; 2019;12(1):1–8. Available from: https://doi.org/10.1186/s13071-019-3455-2

91. Janssen N, Fernandez-Salas I, Díaz González EE, Gaytan-Burns A, Medina-de la Garza CE, Sanchez-Casas RM, et al. Mammalophilic feeding behaviour of Culex quinquefasciatus mosquitoes collected in the cities of Chetumal and Cancun, Yucatán Peninsula, Mexico. Trop Med Int Heal. 2015;20(11):1488–91.

92. Fitzpatrick DM, Hattaway LM, Hsueh AN, Ramos-Niño ME, Cheetham SM. PCR-Based Bloodmeal Analysis of Aedes aegypti and Culex quinquefasciatus (Diptera: Culicidae) in St. George Parish, Grenada. J Med Entomol. 2019;56(4):1170–5.

93. Azmi SA, Das S, Chatterjee S. Seasonal prevalence and blood meal analysis of filarial vector Culex quinquefasciatus in coastal areas of Digha, West Bengal, India. J Vector Borne Dis. 2015;52(3):252–6.

94. Munyao V, Karisa J, Munyao CM, Ngari M, Menza N, Peshu N, et al. Surveillance of culicine mosquitoes in six villages of taita-taveta county, Kenya, with host determinations from blood-fed females. J Med Entomol. 2020;57(6):1972–82.

95. Hopken MW, Reyes-Torres LJ, Scavo N, Piaggio AJ, Abdo Z, Taylor D, et al. Temporal and spatial blood feeding patterns of urban mosquitoes in the san juan metropolitan area, Puerto Rico. Insects. 2021;12(2):1–21.

96. Eastwood G, Cunningham AA, Kramer LD, Goodman SJ. The vector ecology of introduced Culex quinquefasciatus populations, and implications for future risk of West Nile virus emergence in the Galápagos archipelago. Med Vet Entomol. 2019;33(1):44–55.

97. Stenn T, Peck KJ, Rocha Pereira G, Burkett-Cadena ND. Vertebrate Hosts of Aedes aegypti, Aedes albopictus, and Culex quinquefasciatus (Diptera: Culicidae) as Potential Vectors of Zika Virus in Florida. J Med Entomol. 2019;56(1):10–7.

98. Estrada-Franco JG, Fernández-Santos NA, Adebiyi AA, López-López M de J, Aguilar-Durán JA, Hernández-Triana LM, et al. Vertebrate-aedes aegypti and Culex quinquefasciatus (diptera)-arbovirus transmission networks: Non-human feeding revealed by meta-barcoding and nextgeneration sequencing. PLoS Negl Trop Dis. 2020;14(12):1–22.

99. Abella-Medrano CA, Ibáñez-Bernal S, Carbó-Ramírez P, Santiago-Alarcon D. Blood-meal preferences and avian malaria detection in mosquitoes (Diptera: Culicidae) captured at different land use types within a neotropical montane cloud forest matrix. Parasitol Int [Internet]. Elsevier; 2018;67(3):313–20. Available from: https://doi.org/10.1016/j.parint.2018.01.006

100. Egizi AM, Farajollahi A, Fonseca DM. Diverse host feeding on nesting birds may limit early-season west nile virus amplification. Vector-Borne Zoonotic Dis. 2014;14(6):447–53.

101. McPhatter LP, Su T, Williams G, Cheng ML, Dhillon M, Gerry AC. Host-Feeding Patterns of Culex stigmatosoma (Diptera: Culicidae) in Southern California. J Med Entomol. 2017;54(6):1750–7.

102. Friesen KM, Johnson GD. Evaluation of methods for collecting blood-engorged mosquitoes from habitats within a wildlife refuge. J Am Mosq Control Assoc. 2013;29(2):102–7.

103. Reisen WK, Lothrop HD, Thiemann T. Host selection patterns of Culex tarsalis (Diptera: Culicidae) at wetlands near the Salton Sea, Coachella Valley, California, 1998-2002. J Med Entomol. 2013;50(5):1071–6.

104. Thiemann TC, Wheeler SS, Barker CM, Reisen WK. Mosquito host selection varies seasonally with host availability and mosquito density. PLoS Negl Trop Dis. 2011;5(12).

105. Kent R, Juliusson L, Weissmann M, Evans S, Komar N. Seasonal blood-feeding behavior of culex tarsalis (Diptera: culicidae) in weld County, Colorado, 2007. J Med Entomol. 2009;46(2):380–90.

106. Molaei G, Andreadis TG, Armstrong PM, Diuk-Wasser M. Host-feeding patterns of potential mosquito vectors in connecticut, USA: Molecular analysis of bloodmeals from 23 species of Aedes, Anopheles, Culex, Coquillettidia, Psorophora, and Uranotaenia. J Med Entomol. 2008;45(6):1143–51.

107. Reinhold JM, Halbert E, Roark M, Smith SN, Stroh KM, Siler CD, et al. The role of Culex territans mosquitoes in the transmission of Batrachochytrium dendrobatidis to amphibian hosts. Parasites and Vectors [Internet]. BioMed Central; 2023;16(1):1–10. Available from: https://doi.org/10.1186/s13071-023-05992-x

108. Bartlett-Healy K, Crans W, Gaugler R. Vertebrate hosts and phylogenetic relationships of amphibian trypanosomes from a potential invertebrate vector, Culex territans Walker (Diptera: Culicidae). J Parasitol. 2009;95(2):381–7.

109. Evangelista E, Medeiros-Sousa AR, Ceretti-Junior W, Oliveira-Christe R, Wilk-da-Silva R, Duarte AMR de C, et al. Relationship between vertical stratification and feeding habits of mosquito (Diptera: Culicidae) assemblages collected in conservation units in the green belt of the city of São Paulo, Brazil. Acta Trop. 2021;221(June).
