# Supplementary material for: Meta-analyses of Culex blood-meals indicates strong regional effect on feeding patterns
Source: PLoS Negl Trop Dis. 2025 Jan 24;19(1):e0012245. doi: 10.1371/journal.pntd.0012245 (PMC11785302; doi:10.1371/journal.pntd.0012245)
Supplement: S3 Table — The table shows pairwise chi-square comparisons of all included realms per species. Chi-squared values and P values are shown for each comparison. All P values associated with significant differences are indicated in bold. (DOCX) [file pntd.0012245.s013.docx]

**S3 Table. Pairwise chi-square comparisons of all included realms per species.** Chi-squared values and P values are shown for each comparison. All P values associated with significant differences are indicated in bold. P values that were adjusted for multiple testing using the Bonferroni method are shown in the corrected P value column

| **Species** | **Realm comparisons** | **χ2** | **P value** | **Corrected P value** |
| --- | --- | --- | --- | --- |
| *Culex tritaeniorhynchus* | Afrotropical vs Indomalayan | 23.438 | **8.138e-06** | **0.000187** |
|  | Afrotropical vs Palearctic | 90.303 | **0.0004998** | **0.0115** |
|  | Indomalayan vs Palearctic | 90.452 | **<2.2e-16** | **<5.06e-15** |
| *‘Culex pipiens* pooled’ | Afrotropical vs Australasian | 81.604 | **<2.2e-16** | **<5.06e-15** |
|  | Afrotropical vs Nearctic | 129.49 | **<2.2e-16** | **<5.06e-15** |
|  | Afrotropical vs Neotropical | 71.265 | **<2.288e-15** | **<5.2624e-14** |
|  | Afrotropical vs Palearctic | 90.303 | **<2.2e-16** | **<5.06e-15** |
|  | Australasian vs Nearctic | 28.772 | **<2.501e-06** | **<0.0000575** |
|  | Australasian vs Neotropical | 92.546 | **<2.2e-16** | **<5.06e-15** |
|  | Australasian vs Palearctic | 22.626 | **<4.831e-05** | **<0.00111** |
|  | Nearctic vs Neotropical | 92.546 | **<2.2e-16** | **<5.06e-15** |
|  | Nearctic vs Palearctic | 7.5948 | 0.055 | 1.265 |
|  | Neotropical vs Palearctic | 73.082 | **9.336e-16** | **2.147e-14** |
| *Culex quinquefasciatus* | Afrotropical vs Australasian | 165.18 | **<2.2e-16** | **<5.06e-15** |
|  | Afrotropical vs Indomalayan | 79.581 | **<2.2e-16** | **<5.06e-15** |
|  | Afrotropical vs Nearctic | 87.256 | **<2.2e-16** | **<5.06e-15** |
|  | Afrotropical vs Neotropical | 118.51 | **<2.2e-16** | **<5.06e-15** |
|  | Australasian vs Indomalayan | 146.42 | **<2.2e-16** | **<5.06e-15** |
|  | Australasian vs Nearctic | 39.409 | **1.422e-08** | **3.270e-7** |
|  | Australasian vs Neotropical | 146.42 | **<2.2e-16** | **<5.06e-15** |
|  | Indomalayan vs Nearctic | 59.62 | **7.088e-13** | **1.630e-11** |
|  | Indomalayan vs Neotropical | 85.81 | **<2.2e-16** | **<5.06e-15** |
|  | Nearctic vs Neotropical | 5.772 | 0.1232 | 2.834 |
