# Supplementary material for: Meta-analyses of Culex blood-meals indicates strong regional effect on feeding patterns
Source: PLoS Negl Trop Dis. 2025 Jan 24;19(1):e0012245. doi: 10.1371/journal.pntd.0012245 (PMC11785302; doi:10.1371/journal.pntd.0012245)
Supplement: Attachment — Submitted filename: Response to reviewers updates editor comments.docx [file pntd.0012245.s015.docx]

**Response to reviewers**

Dear editors,

Thank you for giving us the opportunity to revise our manuscript ‘Meta-analyses of *Culex* blood-meals indicates strong regional effect on feeding patterns’. We greatly appreciate the time and effort the reviewers have put into their comments. We have changed the manuscript according to these comments. These changes can be found in the track-changes version of the manuscript and in the sections in red. Here we describe our responses to the editors comments. Our responses are written in blue.

**Comments**

1. In response to the reviewer question regarding statistical methods it is stated that "No multiple testing correction was applied". Could the others explain their reasoning behind this choice?

We have now updated our analysis to include a multiple testing correction using the Bonferroni method. The raw and corrected P values can be found in Supplementary information 12. We have updated the methods, figure 3, and it’s description to describe which statistical analyses have been done.

**Our changes:**

**Methods**

The feeding patterns of three major vectors, *Cx. tritaeniorhynchus, ‘Cx. pipiens* pooled’, and *Cx. quinquefasciatus* were analysed across different biogeographical realms. To assess the difference in feeding patterns between realms Pearson’s chi-square test was used. We present raw χ2, raw P vales, and corrected P values using the Bonferroni method in Supporting information 12, allowing the reader to choose or perform alternate multiple testing should they see fit.". This analysis was performed with the stats package (v3.6.2) in R.

**Results**

‘Culex pipiens pooled’, Cx. quinquefasciatus, and Cx. tritaeniorhynchus were chosen for further analysis by realm as they are important vector species which had feeding pattern available in three or more realms. ‘Culex pipiens pooled’ had a majority of avian feeds in the Nearctic, Palearctic and Australasian realms. However, for the Afrotropical realm nearly 90% of ‘Culex pipiens pooled’ feeds was recorded on non-human mammals and humans. In the Neotropical realm close to 70% of all blood-meals was recorded on non-human mammals. All of the realms differed significantly in their feeding patterns for ‘Culex pipiens pooled’ except for the Nearctic and Palearctic realm (χ2= 7.59, df = 3, p > 0.05)(Fig 3). Culex quinquefasciatus had a majority of mammal feeds in the Afrotropical realm, human feeds in the Indomalayan realm, and avian feeds in the Australasia and Neotropical realms. In the Nearctic realm there was a similar percentage of avian and mammalian feeding. All realms had significantly different feeding patterns for Culex quinquefasciatus except for the Nearctic and Neotropical realm (χ2= 5.772, df = 3, p > 0.05) (Fig 3). Culex tritaeniorhynchus feeding patterns in the Afrotropical and Indomalayan realms, had a majority of feeds being on non-human mammalian hosts. In the Palearctic realm a majority of avian feeds was observed. All realms had significantly different feeding patterns (Fig 3). In Fig. 3 we present our statistical analysis without multiple testing correction. We included the most commonly used and stringent multiple testing correction method (the Bonferroni method) in the detailed analysis in Supporting information 12. We show that the Bonferroni universal null hypothesis can be rejected, giving further support to our results, and we present raw χ2, raw P vales, and corrected P values in Supporting information 12, allowing the reader to choose or perform alternate multiple testing should they see fit.

**Fig 3. Feeding patterns of ‘Culex pipiens pooled’, Culex quinquefasciatus, and Culex tritaeniorhynchus in different biogeographical realms.** Percentage of blood-meals on amphibian, avian, human, non-human mammal, and reptile hosts are shown for each realm. The total number of blood-meals and total number of publications (blood-meals(publications)) is shown below each bar. ‘Culex pipiens pooled’ consists of the following mosquitoes: Culex pipiens not specified (ns), Culex pipiens molestus, Culex pipiens pallens, Culex pipiens pipiens, Culex pipiens/molestus hybrid. Bars with the same letter above them do not differ significantly (P>0.05) in pairwise comparisons by Chi-square. See Supporting information 12 for raw χ2, raw P vales, and corrected P values using the Bonferroni correction method.  Pairwise comparisons of realms were undertaken for each species; pairwise comparison of realms between species was not undertaken. The numbers in the bar represent the percentages of feeds on each host group for a specific species in that realm. The variation between studies for this analysis is shown in Supporting information 13.

2. molecular methods: what is exactly meant with 'agarose gel diffusion'? Does this mean PCR followed by gel electrophoresis? If yes, agarose gel diffusion is not the conventional way to refer to this. If no, please clarify.

Thank you for the comment. Agarose gel diffusion is a technique that can be used to identify bloodmeals from mosquito midguts. In this technique antigen and antibody are introduced into wells on an agarose gel and allowed to diffuse. After a certain amount of time they leave a precipitate band which can be read to determine the source of the bloodmeal (Crans, 1969; <https://vectorbio.rutgers.edu/publications/An_agar_gel_diffusion_method_for_id_mosq_blood.pdf>). The articles that are included in our database from Philip Samuel et al. (2008) and Azmi et al. (2015) use this agarose gel diffusion method.
