# Supplementary material for: Meta-analyses of Culex blood-meals indicates strong regional effect on feeding patterns
Source: PLoS Negl Trop Dis. 2025 Jan 24;19(1):e0012245. doi: 10.1371/journal.pntd.0012245 (PMC11785302; doi:10.1371/journal.pntd.0012245)
Supplement: Attachment — Submitted filename: Response to reviewers.docx [file pntd.0012245.s014.docx]

**Response to reviews**

Dear editors,

Thank you for giving us the opportunity to revise our manuscript ‘Meta-analyses of *Culex* blood-meals indicates strong regional effect on feeding patterns’. We greatly appreciate the time and effort the reviewers have put into their comments. We have changed the manuscript according to these comments. These changes can be found in the track-changes version of the manuscript and in the sections in red. Here we describe our responses to the reviewers’ comments. Our responses are written in blue.

**Methods**

Reviewer #1: The authors selected the COI gene to compare mosquito phylogeny and feeding patterns. While the COI gene is a commonly used gene for species identification, it may not be the best gene for phylogenetic analyses. A recent study used 709 genes for broad-level mosquito phylogeny and analyzed close to 300,000 records of blood meals from 422 species (Soghigian et al. 2023, Nature Communications). Using a similar approach in which multiple genes are used to reconstruct the mosquito phylogeny is more appropriate and this may impact the associations that are found with feeding preferences.

We agree using multiple genes would be optimal, however this would be suitable for well-studied species only, and we are looking at the full breadth, with a lot of the included *Culex* species in our database only having a COI sequence available such as, *Culex zombaensis, Culex stigmatosoma, Culex restrictor, Culex janitor, Culex globocoxitus, Culex crinicauda*. Or many of them not even having a COI gene sequence such as *Culex cedecei, Culex chrusonotum, Culex hili.* So, whilst our method might not produce the most accurate phylogenetic tree for well-studied species, it does allow us to include as many species as possible (including the above), which we would otherwise not be able to, therefore enabling us to perform a higher resolution analysis than the nature paper. To enhance clarity of our choice we have explained the choice for this gene in more detail in the methods.

**Our changes:**

**Methods – lines 138 - 142**

Multiple sequence alignments were made of the cytochrome c oxidase subunit I (COI) gene. This gene is commonly used for mosquito species identification as it is easy to amplify and has enough variation in its sequence to distinguish between different species (25). The COI gene sequence is therefore available for many different *Culex* species, allowing as many *Culex* species as possible to be included in the phylogeny.

Reviewer #2: statistical analyses - it's unclear if the large set of pairwise comparisons were corrected for multiple comparisons

No multiple testing correction was applied to the P-values on any test in the paper. As it was unclear from our previous description of the statistical methods, we have improved this description. We have clarified the statistical methods in a separate paragraph, see below. We have also included more detail in the description of Figure 3.

**Our changes**:

**Methods – lines 159 - 163**

*Feeding patterns between realms*

The feeding patterns of three major vectors, *Culex tritaeniorhynchus, ‘Cx. pipiens* pooled’, and *Cx. quinquefasciatus* were analysed across different biogeographical realms. To assess the difference in feeding patterns between realms Pearson’s chi-square test was used, no multiple testing correction was applied to the P values. This analysis was performed with the stats package (v3.6.2) in R.

**Results (Feeding patterns across biogeographical realms) – lines 232 - 245**

**Fig 3. Feeding patterns of *‘Culex pipiens* pooled’, *Culex quinquefasciatus*, and *Culex tritaeniorhynchus* in different biogeographical realms.** Percentage of blood-meals on amphibian, avian, human, non-human mammal, and reptile hosts are shown for each realm. The total number of blood-meals and total number of publications (blood-meals(publications)) is shown below each bar. ‘*Culex pipiens* pooled’ consists of the following mosquitoes: *Culex pipiens* not specified (ns)*, Culex pipiens molestus, Culex pipiens pallens, Culex pipiens pipiens, Culex pipiens/molestus* hybrid*.* Significance groups of Chi-square pairwise comparisons are shown in the figure. No multiple corrections were applied to the pairwise comparisons. Superscript letters above the bars indicate significance groups. If two bars share the same letter they do not differ significantly (p>0.05). If two bars share a different letter they do differ significantly (p<0.05). Subscript numbers separate different pairwise comparisons within the three vector species (i.e. there were no pairwise comparisons between different mosquito species). The numbers in the bar represent the percentages of feeds on each host group for a specific species in that realm. See Supporting information 12 for the full list of pairwise comparisons and P values. The variation between studies for this analysis is shown in Supporting information 13.

Reviewer #3: -The hypothesis of the study is confused because authors mix medical importance in a overall genus study that include species without medical importance. I recomend define two hypothesis: one within the genus analysis and another focus on the medical important species.

We have adjusted our introduction and separated the aims of our analyses; the adjusted introduction is shown below. To enhance the clarity of which species are medically important we have highlighted *Culex* species that are competent vectors for several different pathogens in bold in Figure 2, as the combination of vector competence and certain feeding patterns could indicate whether a species could play a role in a potential outbreak. Additionally, we have expanded in the discussion why we believe it is important to focus on species that are currently not medically important as well.

**Our changes:**

**Introduction – lines 85 - 92**

Here, we analyse the data published over the last 15 years describing *Culex* blood-feeding in the field. We first look at the whole genus level and then focus in on three important *Culex* vectors. For the whole *Culex* genus we analyse whether there is a pattern between phylogenetic relatedness and feeding patterns. For three important vector species we assess the relationship between biogeographical realm and feeding patterns. Overall, this analysis enables a global insight into the feeding patterns of *Culex* mosquitoes and thus provides higher-level data for risk assessments to specific hosts, and to specific regions, which will inform ongoing efforts, particularly in Europe (18,19), to mitigate the spread of *Culex*-borne viruses (e.g. West Nile virus and Usutu virus) (20).

**Discussion – lines 271 - 277**

There were several other species which had feeding patterns that included both avian and mammalian hosts but are not well known WNV vectors (*Cx. erythrothorax, Cx. nigripalus, Cx. orbostiensis, Cx. orientalis, Cx. perexiguus, Cx. restrictor, Cx. sitiens*). These species could potentially be implicated in a future outbreak, but perhaps this has not been observed or the species hasn’t been in contact with the virus yet. Thus, being aware of the feeding patterns of mosquito species which are currently not well-known vectors is still relevant to assess future risks.

**Fig 2. Tanglegram of a phylogenetic tree based on the cytochrome c oxidase subunit I (COI) gene of 57 *Culex* (*Cx.*) mosquitoes compared to a hierarchical clustering tree of *Culex* mosquito feeding patterns.** The phylogenetic tree (shown on the left) was generated using IQ-TREE with 1000 ultrafast bootstrap alignments. Node support (bootstrap values) is indicated using coloured circles in the phylogenetic tree. If the node support was less than 50, no circle was placed on the clade. The labels of species that are known vectors for several medically relevant pathogens are bold in the phylogenetic tree (31).The hierarchical clustering tree (shown on the right) was created in R using the *hclust* function. The phylogenetic and feeding tree are connected between the same species. The branch length of the trees represents the distance between clusters. No clusters were found that were the same in both trees. Next to the feeding dendrogram the percentage of feeds on each host group is presented per *Culex* species. The *Chaoboridae* family was chosen as the outgroup. The number of blood-meals and publications per *Culex* species are found in Supporting information 4. For each species the variation in number of blood-meals on major host groups per study is shown in Supporting information 10. The same feeding proportions are lined up with the phylogeny, rather than clustered and rearranged, in Supporting information 11.

-ref40: it should be in the introduction

We have updated our introduction to now include this reference.

**Our changes:**

**Introduction – lines 77 - 80**

These realms represent an area that has a similar plant an animal distribution and that experienced a distinct evolutionary history and climate (16). Previous analyses have shown that mosquito feeding patterns are associated with major continental drift events (17).

-lack of consistency in representing pipiens and molestus forms.

Thank you for pointing this out. The inconsistencies have been changed.

Culex quinquefasciatus is part of the Cx. pipiens complex and authors may use Cx. pipiens s.l. and Cx. pipiens s.s. to differenciate or include Cx. quinquefasciatus in the discussion or to represent an agregate analysis within the species or among the subspecies/forms of Cx. pipiens.

For our analysis we chose to include the following species in ‘*Culex pipiens* pooled’ : *Culex pipiens* not specified (ns)*, Culex pipiens molestus, Culex pipiens pallens, Culex pipiens pipiens, Culex pipiens/molestus* hybrid*.* We did not include *quinquefasciatus* in this group as this species is easily distinguishable from other *Culex pipiens* mosquitoes (ECDC, 2020; <https://www.ecdc.europa.eu/en/infectious-disease-topics/related-public-health-topics/disease-vectors/facts/mosquito-factsheets/culex-pipiens>). This distinction was made in 39 of the papers included in this analysis (Muturi et al, 2008; DOI: 10.1007/s00436-008-0914-7, Johansen et al., 2009; DOI: 10.1603/033.046.0527; Stenn et al., 2019; DOI: 10.1111/mve.12329, Santos et al., 2019; DOI: 10.1371/journal.pone.0212517, Janssen et al., 2015; DOI: 10.1111/tmi.12587). We have also changed the abbreviation we use for *Culex pipiens* not specified to enhance clarity. The improved explanation of *Culex pipiens* pooled can be found below.

**Our changes:**

**Methods – lines 119 - 123**

If the specific form of *Culex pipiens* was not specified, the data was categorized under *Culex pipiens* not specified (ns)*.* Furthermore, for our analysis *Culex pipiens* (ns)*, Culex pipiens molestus, Culex pipiens pallens, Culex pipiens pipiens, Culex pipiens/molestus* hybrid were clustered as ‘*Culex pipiens* pooled’.

-line 48-49: I will remove this sentence because most mosquito genus with vector competence occur across multiple continents and habitats. It is unlikely that these two references claim that Culex genus is special because their species occur in both tropical and temperate regions.

Thanks for the comment, the sentence has been removed.

-lines 50-52: Some concepts in this sentence are being miss use. Reservoir host is an important concept for the dynamic of some diseases when the etiological agent can survive in a residual way within this vertebrate host, but it is normally not used for vectors.

Thanks for the comment, reservoir was mistakenly written twice. This has now been correct so that it only refers to the vertebrate.

**Our changes:**

**Introduction – lines 49 - 51**

Many *Culex* species can act as vectors, transmitting pathogens between reservoir hosts, or transmitting from reservoir hosts to dead-end hosts which, in some cases, includes humans.

Bridge vector is a very specific concept for WNV transmission and I should avoid the generalization.

This is incorrect, the term bridge vector is used for other viruses e.g. USUV (Clé et al., 2019; DOI: <https://doi.org/10.1017/S0950268819001213> ) and YFV (Pereira-dos-Santos et al., 2020; DOI: [10.3390/pathogens9040266](https://doi.org/10.3390/pathogens9040266)).

-The bibliography search require more detail. How many articles were found, how they were defined as valid. I recomend folow some guidelines from PICOS or SPIDER methods to improve the description of the data collection.

Thank you for the comment. We assume the reviewer believes the data table to be incomplete. We followed up our search with more detailed search terms and redid the screening to ensure we included as many records as possible. The updated database can be found in Supporting information 2. We have created a more detailed description of our bibliography search in our methods which can be seen in red below. In Supporting information 1 you will find a PRISMA scheme which describes our literature search in more detail. Now a total of 109 records are included in our database.

**Our changes:**

**Methods – lines 97 - 112**

Culex blood-feeding studies were collected using the PubMed database. The combination of keywords: “Culex”, “Cx.” “blood meal”, “bloodmeal”, “blood-meal”, “mosquito”, “host-species”, “host”, and “feeding” was used to find relevant publications. For several species, where data were lacking, additional searches were performed included the full species name. No additional records were found using this strategy. English literature from 2008-2023 was included in this review. This 15-year period was chosen to focus on recent feeding patterns of Culex mosquitoes. A total of 288 records were identified for screening. All abstracts were screened and all records that did not describe blood feeding of Culex mosquitoes were excluded. For the remaining 153 records the full text was analysed. Papers were included in our analysis if they specified the number of blood-meals and if they utilized molecular methods to confirm the blood-meal source. Molecular methods that were accepted for inclusion were: PCR, mNGS, ELISA, antiserum, and agarose gel diffusion. Studies performed in zoos were included, as they are a location where mosquitoes are free to choose or not choose for a specific host under field conditions. Review studies were not included. Studies which analysed the presence of Culex species in animal traps and laboratory-based blood-feeding studies were excluded. Additionally, studies which introduced mosquitoes from the lab to the field conditions were excluded as well. The full overview of our screening process can be found in Supporting information 1.

**Supporting information 1**

Figure 1 PRISMA flowchart of selection and inclusion process for the meta-analysis of Culex blood-meal studies from the last 15 years. A total of 288 records were screened from PubMed. After the screening process 109 records were included in the meta-analysis.

Which species require further analysis beyond genus search?

This was attempted but no additional records were found.

Which molecular methods were accepted?

The methods are now included in the database, which can be found at Supplementary information 2. The methods that are included are: PCR, mNGS, ELISA, antiserum, and agarose gel diffusion. To enhance clarity, we have improved our description of the methods by describing which molecular methods which were accepted in our analysis.

**Our changes:**

**Methods – lines 105 - 107**

Papers were included in our analysis if they specified the number of blood-meals and if they utilized molecular methods to confirm the blood-meal source. Molecular methods that were accepted for inclusion were: PCR, mNGS, ELISA, antiserum, and agarose gel diffusion.

-Some statistical analysis are described but not presented.

We have added additional analyses and improved our description of the statistical methods.

**Our changes:**

**Methods – lines 151 - 163**

For the 57 species that had COI genes available on NCBI a tanglegram was created which compared Culex phylogeny to a tree based on the feeding patterns of the same mosquito. For this tanglegram the Baker’s gamma index (BGI) (29) and the Pearson correlation (30) was calculated, which give an estimation of the association of the two dendrograms compared in the tanglegram. To determine the statistical significance of the BGI the labels for one of the trees were shuffled and the BGI was calculated again (n = 999). This tanglegram and the statistical tests were done with the dendextend package in R (30).

*Feeding patterns between realms*

The feeding patterns of three major vectors, *Cx. tritaeniorhynchus, ‘Cx. pipiens* pooled’, and *Cx. quinquefasciatus* were analysed across different biogeographical realms. To assess the difference in feeding patterns between realms Pearson’s chi-square test was used, no multiple testing correction was applied to the P values. This analysis was performed with the stats package (v3.6.2) in R.

--------------------

Reviewer #1: In Figure 2, the x-axis is missing for the mosquito phylogeny and there is no branch support included. There also appears to be a typo in the legend: “The height of the trees represents the distance between clusters”. Not the height of the tree but the branch length indicates genetic distance.

We have now added an x-axis for the mosquito phylogeny and feeding pattern tree. We have also included branch support values. The description of the figure has been improved.

**Our changes:**

**Results – lines 203 - 215**

**Fig 2. Tanglegram of a phylogenetic tree based on the cytochrome c oxidase subunit I (COI) gene of 57 *Culex* (*Cx.*) mosquitoes compared to a hierarchical clustering tree of *Culex* mosquito feeding patterns.** The phylogenetic tree (shown on the left) was generated using IQ-TREE with 1000 ultrafast bootstrap alignments. Node support (bootstrap values) is indicated using coloured circles in the phylogenetic tree. If the node support was less than 50, no circle was placed on the clade. The labels of species that are known vectors for several medically relevant pathogens are bold in the phylogenetic tree (31).The hierarchical clustering tree (shown on the right) was created in R using the *hclust* function. The phylogenetic and feeding tree are connected between the same species. The branch length of the trees represents the distance between clusters. No clusters were found that were the same in both trees. Next to the feeding dendrogram the percentage of feeds on each host group is presented per *Culex* species. The *Chaoboridae* family was chosen as the outgroup. The number of blood-meals and publications per *Culex* species are found in Supporting information 4. For each species the variation in number of blood-meals on major host groups per study is shown in Supporting information 10. The same feeding proportions are lined up with the phylogeny, rather than clustered and rearranged, in Supporting information 11.

The lack of more detailed information on collection location, host species, and host abundance prevents the current study from increasing the understanding of the underlying drivers of host-associations beyond describing the associations.

We have added more detailed information on collection location (indoor/outdoor), collection method, and land use type in our database. Our database also contains information on the host species to the level described in literature, which is most common to species level. Most of the included publications did not contain information on host abundance, however as this would give us a better insight into why certain mosquitoes choose to feed on certain hosts this would be beneficial information to have. This is why we describe the need for host abundance data in the discussion. We have analysed the additional data that was added to our database and the figures are included in Supporting information 6, 7, 8.

**Our changes:**

**Methods – lines 114 – 119**

From the articles that were included the following information was extracted: PMID, Title, Authors, Publication year, DOI, Mosquito species, Mosquito location (country), Biogeographical realm, Host (to level described in literature), Host (major host group: amphibian, avian, human, non-human mammalian, and reptilian), Number of bloodmeals, Total number of bloodmeals (in that specific study), Collection method, Bloodmeal analysis method, Land use type (of where study was done), Indoor/outdoor collections.

**Methods – lines 165 - 169**

*Study characteristics*

For every included *Culex* species the percentage of feeds across different land use types was analysed. These results are described in Supporting information 6. For *Cx. tritaeniorhynchus, ‘Cx. pipiens* pooled’, and *Cx. quinquefasciatus* the different collection methods and collection locations (indoor/outdoor) were analysed as well. These results are presented in Supporting information 7 and 8 respectively.

**Discussion – lines 323 - 343**

Land use could be an important factor in determining host utilization of these mosquitoes. Geographically distinct populations of mosquitoes can have different host preferences (42). Urbanization affects feedings patterns as well, with Cx. pipiens and Cx. quinquefasciatus having increased levels of human feeding in urban areas (10). Where available we collected the data on the different land use types included in the studies of this meta-analysis (Supporting information 2). We summarized the feeding patterns of each Culex species on the major host groups across different land use types in Supporting information 6. Additionally, whether the collection occurs inside or outside can have an effect, with inside collections having a significantly higher percentage of human blood feeding compared to collection that just occurred outside (43). If specified, the collection location (indoor/outdoor) and the collection method was also included in our database as well (Supporting information 2). We analysed the different collection locations and collection method for ‘Culex pipiens pooled’, Culex quinquefasciatus, and Culex tritaeniorhynchus in Supporting information 7 and 8. These figures show that for each of these species relatively similar collection locations and methods were used. Most of the included studies did include some form of description of land use types, however the descriptions did greatly vary in the amount of detail and often lacked a clear quantification of land-use type. This made it challenging to draw any conclusions from the land use data. Future studies should aim to include a description of land use types in a 1 km radius around the collection point of the bloodfed mosquito. They should also give a clear reason why they classify a certain area as urban, rural, or natural. If the data are not available, studies should provide additional information and/or supplementary pictures of the location of the mosquito-collections. Specific information on whether these collections were performed indoor or outdoor should also be given.

**Supporting information 6**

Figure 1 *Culex* feeding patterns across different land use types. Each figure shows the percentage of blood-meals taken on five major host groups: amphibian, avian, human, non-human mammal, reptile per mosquito species. Where data was available land use from different studies was classified into six different types: mixed (mosquitoes from multiple types of land use were collected in this study), natural (land that is not modified for human use, such as wetlands, forests, grasslands, etc.), not specified (if no detailed description was given of land use the study was classified as not specified), rural (if this was described in the original publication, or the publication described low population density, small settlements, farming, and agriculture), urban (if this was described in the original publication, or the publication described a major city), and zoo. The title of each graph shows the mosquito species (Number of blood-meals (number of studies)).

**Supporting information 7**

Figure 1 Percentage of blood-meals per major host group: amphibian, avian, human, non-human mammal, and reptile, that were collected using different methods, or combinations of methods. This analysis was done for ‘Culex pipiens pooled’, Culex quinquefasciatus, Culex tritaeniorhynchus. ‘Culex pipiens pooled’ describes a pool of species that contains: Culex pipiens not specified (ns), Culex pipiens molestus, Culex pipiens pallens, Culex pipiens pipiens, Culex pipiens/molestus hybrid. The title of each graph shows the mosquito species (Number of blood-meals (number of studies)). In each bar the exact number of blood-meals collected using the different methods is shown.

**Supporting information 8**

Figure 1 Percentage of blood-meals collected indoor, mixed (=indoor and outdoor), not specified, and outdoor per major host group: amphibian, avian, human, non-human mammal, and reptile. This analysis was done for ‘Culex pipiens pooled’, Culex quinquefasciatus, Culex tritaeniorhynchus. ‘Culex pipiens pooled’ describes a pool of species that contains: Culex pipiens not specified (ns), Culex pipiens molestus, Culex pipiens pallens, Culex pipiens pipiens, Culex pipiens/molestus hybrid. The title of each graph shows the mosquito species (Number of blood-meals (number of studies)). In each bar the number of blood-meals is shown for each collection location.

Moreover, the lack of data on accurately identified species/subspecies/biotypes, which are known to have different feeding preferences, prevents a more detailed analysis of the broader feeding patterns of these lower-level groups that are important for risk assessments. I appreciate that the authors acknowledge these limitations (and provide recommendations for how to improve future studies), but, unfortunately, this does limit the overall impact of the study.

We agree that more data on accurately identified species/subspecies/biotypes would allow for a more detailed analysis. We aimed to shed light on these differences by where possible showing separate feeding patterns for separate biotypes, *Culex pipiens pipiens, Culex pipiens molestus* etc. However, as the number of blood-meals collected for these specific biotypes were low we decided to pool some of these into ‘Culex pipiens pooled’ which still allowed us to gain an insight into the feeding patterns of *Culex pipiens* mosquitoes as a whole.

Reviewer #2: - The analysis leaves out important potential confounders such as collection site type (eg rural/urban/natural) and collection methods, which could be driving the observed results.

We have added more detailed information on collection location (indoor/outdoor), collection method, and land use type in our database. Our database also contains information on the host species to the level described in literature, which is most common to species level. Most of the included publications did not contain information on host abundance, however as this would give us a better insight into why certain mosquitoes choose to feed on certain hosts this would be beneficial information to have. This is why we describe the need for host abundance data in the discussion. We have analysed the additional data that was added to our database and the figures are included in Supporting information 6, 7, 8.

**Our changes:**

**Methods – lines 114 – 119**

From the articles that were included the following information was extracted: PMID, Title, Authors, Publication year, DOI, Mosquito species, Mosquito location (country), Biogeographical realm, Host (to level described in literature), Host (major host group: amphibian, avian, human, non-human mammalian, and reptilian), Number of bloodmeals, Total number of bloodmeals (in that specific study), Collection method, Bloodmeal analysis method, Land use type (of where study was done), Indoor/outdoor collections.

**Methods – lines 165 - 169**

*Study characteristics*

For every included *Culex* species the percentage of feeds across different land use types was analysed. These results are described in Supporting information 6. For *Cx. tritaeniorhynchus, ‘Cx. pipiens* pooled’, and *Cx. quinquefasciatus* the different collection methods and collection locations (indoor/outdoor) were analysed as well. These results are presented in Supporting information 7 and 8 respectively.

**Discussion – lines 323 - 343**

Land use could be an important factor in determining host utilization of these mosquitoes. Geographically distinct populations of mosquitoes can have different host preferences (42). Urbanization affects feedings patterns as well, with Cx. pipiens and Cx. quinquefasciatus having increased levels of human feeding in urban areas (10). Where available we collected the data on the different land use types included in the studies of this meta-analysis (Supporting information 2). We summarized the feeding patterns of each Culex species on the major host groups across different land use types in Supporting information 6. Additionally, whether the collection occurs inside or outside can have an effect, with inside collections having a significantly higher percentage of human blood feeding compared to collection that just occurred outside (43). If specified, the collection location (indoor/outdoor) and the collection method was also included in our database as well (Supporting information 2). We analysed the different collection locations and collection method for ‘Culex pipiens pooled’, Culex quinquefasciatus, and Culex tritaeniorhynchus in Supporting information 7 and 8. These figures show that for each of these species relatively similar collection locations and methods were used. Most of the included studies did include some form of description of land use types, however the descriptions did greatly vary in the amount of detail and often lacked a clear quantification of land-use type. This made it challenging to draw any conclusions from the land use data. Future studies should aim to include a description of land use types in a 1 km radius around the collection point of the bloodfed mosquito. They should also give a clear reason why they classify a certain area as urban, rural, or natural. If the data are not available, studies should provide additional information and/or supplementary pictures of the location of the mosquito-collections. Specific information on whether these collections were performed indoor or outdoor should also be given.

**Supporting information 6**

Figure 1 *Culex* feeding patterns across different land use types. Each figure shows the percentage of blood-meals taken on five major host groups: amphibian, avian, human, non-human mammal, reptile per mosquito species. Where data was available land use from different studies was classified into six different types: mixed (mosquitoes from multiple types of land use were collected in this study), natural (land that is not modified for human use, such as wetlands, forests, grasslands, etc.), not specified (if no detailed description was given of land use the study was classified as not specified), rural (if this was described in the original publication, or the publication described low population density, small settlements, farming, and agriculture), urban (if this was described in the original publication, or the publication described a major city), and zoo. The title of each graph shows the mosquito species (Number of blood-meals (number of studies)).

**Supporting information 7**

Figure 1 Percentage of blood-meals per major host group: amphibian, avian, human, non-human mammal, and reptile, that were collected using different methods, or combinations of methods. This analysis was done for ‘Culex pipiens pooled’, Culex quinquefasciatus, Culex tritaeniorhynchus. ‘Culex pipiens pooled’ describes a pool of species that contains: Culex pipiens not specified (ns), Culex pipiens molestus, Culex pipiens pallens, Culex pipiens pipiens, Culex pipiens/molestus hybrid. The title of each graph shows the mosquito species (Number of blood-meals (number of studies)). In each bar the exact number of blood-meals collected using the different methods is shown.

**Supporting information 8**

Figure 1 Percentage of blood-meals collected indoor, mixed (=indoor and outdoor), not specified, and outdoor per major host group: amphibian, avian, human, non-human mammal, and reptile. This analysis was done for ‘Culex pipiens pooled’, Culex quinquefasciatus, Culex tritaeniorhynchus. ‘Culex pipiens pooled’ describes a pool of species that contains: Culex pipiens not specified (ns), Culex pipiens molestus, Culex pipiens pallens, Culex pipiens pipiens, Culex pipiens/molestus hybrid. The title of each graph shows the mosquito species (Number of blood-meals (number of studies)). In each bar the number of blood-meals is shown for each collection location.

- As noted in the main comments, the annotation for Figure 2 needs to be more clearly described

The description of the figure has been improved.

**Our changes:**

**Results – lines 202 - 214**

**Fig 2. Tanglegram of a phylogenetic tree based on the cytochrome c oxidase subunit I (COI) gene of 57 *Culex* (*Cx.*) mosquitoes compared to a hierarchical clustering tree of *Culex* mosquito feeding patterns.** The phylogenetic tree (shown on the left) was generated using IQ-TREE with 1000 ultrafast bootstrap alignments. Node support (bootstrap values) is indicated using coloured circles in the phylogenetic tree. If the node support was less than 50, no circle was placed on the clade. The labels of species that are known vectors for several medically relevant pathogens are bold in the phylogenetic tree (31).The hierarchical clustering tree (shown on the right) was created in R using the *hclust* function. The phylogenetic and feeding tree are connected between the same species. The branch length of the trees represents the distance between clusters. No clusters were found that were the same in both trees. Next to the feeding dendrogram the percentage of feeds on each host group is presented per *Culex* species. The *Chaoboridae* family was chosen as the outgroup. The number of blood-meals and publications per *Culex* species are found in Supporting information 4. For each species the variation in number of blood-meals on major host groups per study is shown in Supporting information 10. The same feeding proportions are lined up with the phylogeny, rather than clustered and rearranged, in Supporting information 11.

Reviewer #3: -I notice that some articles that are within the criteria described are not included in the Table S1. It may be important have the journal information. I notice that some articles in Parasites& vectors are missing, but it is difficult to check for missing these information.

We have followed up our search with more detailed search terms and redid the screening to ensure we included as many records as possible. The updated database can be found in Supporting information 2. We have added the journal information of each included publication.

-Table showing number of article per species should be presented. All articles should be cited. Include number individuals and/or populations per species may be important to verify the robustness of the data per species.

We have created a table showing the number of blood-meals and publications per species. The citations are included in this table as well. Supporting information 4 contains both the table describing number of blood-meals, number of publications, and corresponding references as well as the fully written out reference list.

**Supporting information 4**

Table 1 Number of blood-meals and number of publications recorded per Culex species. For each species the references describing the bloodmeals are shown.

| **Mosquito species** | **Number of blood-meals** | **Number of publications** | **Reference** |
| --- | --- | --- | --- |
| **Culex amazonensis** | 1 | 1 | (1) |
| **Culex annulioris** | 59 | 1 | (2) |
| **Culex annulirostris** | 1870 | 3 | (3),(4),(5) |
| **Culex antennatus** | 3 | 1 | (6) |
| **Culex acinus** | 15 | 1 | (7) |
| **Culex atratus** | 208 | 1 | (8) |
| **Culex australicus** | 8 | 2 | (3),(5) |
| **Culex bahamensis** | 1 | 1 | (9) |
| **Culex bitaeniorhynchus** | 2 | 1 | (10) |
| **Culex caudelli** | 2 | 1 | (1) |
| **Culex cedecei** | 667 | 2 | (11), (12) |
| **Culex chidesteri** | 7 | 2 | (13), (7) |
| **Culex chrysonotum** | 2 | 1 | (14) |
| **Culex coronator** | 53 | 4 | (13), (15), (16), (14) |
| **Culex crinicauda** | 1 | 1 | (5) |
| **Culex decens** | 3 | 1 | (6) |
| **Culex declarator** | 18 | 1 | (1) |
| **Culex dolosus** | 8 | 1 | (7) |
| **Culex eduardoi** | 10 | 1 | (7) |
| **Culex erraticus** | 1461 | 9 | (17), (18), (15), (19), (20), (21), (22), (23), (24) |
| **Culex erythrothorax** | 88 | 2 | (25), (26) |
| **Culex gelidus** | 44 | 3 | (5), (27), (28) |
| **Culex globocoxitus** | 13 | 1 | (5) |
| **Culex halifaxii** | 1 | 1 | (3) |
| **Culex hilli** | 4 | 1 | (3) |
| **Culex hortensis** | 8 | 1 | (29) |
| **Culex interrogator** | 18 | 1 | (14) |
| **Culex intrincatus** | 1 | 1 | (30) |
| **Culex iolambdis** | 261 | 1 | (31) |
| **Culex janitor** | 5 | 1 | (9) |
| **Culex lactator** | 2 | 1 | (14) |
| **Culex lahilei** | 1 | 1 | (7) |
| **Culex melanoconion sp.** | 42 | 3 | (13), (30), (9) |
| **Culex microculex sp.** | 3 | 1 | (30) |
| **Culex modestus** | 107 | 5 | (32), (33), (34), (35), (36) |
| **Culex mollis** | 1 | 1 | (30) |
| **Culex naivei** | 17 | 1 | (37) |
| **Culex neavei** | 6 | 1 | (38) |
| **Culex nigripalpus** | 877 | 10 | (13), (39), (18), (15), (40), (16),(14), (1), (9), (41) |
| **Culex orbostiensis** | 4 | 1 | (3) |
| **Culex orientalis** | 23 | 2 | (42), (10) |
| **Culex peccator** | 13 | 1 | (17) |
| **Culex pedroi** | 1 | 1 | (1) |
| **Culex perexiguus** | 127 | 5 | (33), (34), (43), (44), (45) |
| **Culex pilosus** | 173 | 2 | (8), (21) |
| **Culex pipiens molestus** | 267 | 8 | (46), (3), (47), (48), (49), (50), (7), (51) |
| **Culex pipiens molestus hybrid** | 44 | 3 | (52), (48), (51) |
| **Culex pipiens ns** | 5287 | 36 | (53), (54), (55), (29), (56), (32), (57), (52), (58), (49), (59), (33), (60), (34), (61), (62), (63), (64), (43), (44), (65), (66), (67), (68), (36), (45), (69), (70), (71), (72), (73), (25), (74), (75), (37), (76) |
| **Culex pipiens pallens** | 316 | 3 | (47), (77), (78) |
| **Culex pipiens pipiens** | 594 | 6 | (48), (79), (50), (43), (51), (24) |
| **Culex pipiens quinquefasciatus hybrid** | 52 | 4 | (46), (52), (7), (24) |
| **Culex poicilipes** | 32 | 3 | (2), (6), (37) |
| **Culex pullus** | 28 | 2 | (3), (5) |
| **Culex quinquefasciatus** | 10969 | 40 | (17), (80), (46), (81), (13), (3), (2), (82), (52), (83), (39), (5), (84), (15), (85), (40), (86), (16), (14), (87), (7), (88), (89), (28), (90), (21), (91), (30), (9), (6), (92), (93), (41), (94), (95), (96), (24), (26), (97), (98) |
| **Culex restrictor** | 2 | 1 | (20) |
| **Culex restuans** | 520 | 11 | (17), (55), (99), (52), (79), (58), (100), (18), (63), (64), (72) |
| **Culex salinarius** | 93 | 4 | (16), (72), (73), (24) |
| **Culex sitiens** | 121 | 4 | (3), (5), (43), (90) |
| **Culex spissipes** | 1 | 1 | (1) |
| **Culex stigmatosoma** | 660 | 4 | (14), (101), (70), (26) |
| **Culex taeniopus** | 51 | 2 | (14), (9) |
| **Culex tarsalis** | 2227 | 10 | (60), (102), (103), (69), (70), (104), (105), (25), (74), (26) |
| **Culex territans** | 350 | 8 | (17), (18), (62), (63), (106), (68), (107), (108) |
| **Culex theileri** | 187 | 4 | (33), (34), (43), (44) |
| **Culex tritaeniorhynchus** | 1865 | 6 | (10), (38), (27), (28), (6), (76) |
| **Culex univitattus** | 53 | 4 | (53), (6), (94), (37) |
| **Culex vansomerini** | 4 | 1 | (37) |
| **Culex vaxus** | 6 | 1 | (109) |
| **Culex zombaensis** | 2 | 1 | (54) |

-Figure 1: Cx. pipiens branch is odd. How do you have phylogeny from COI with Cx. pipiens pooled?

A non-specified form of Culex pipiens was used for this branch in the phylogeny. However, we agree that this is not a good representation, so the branch has been removed from the phylogeny.

Missing bootstraps information in Tree and other overall statistical analysis results

We have added the bootstrap values and Pearson correlation to the figure.

**Our changes:**

**Results – lines 202 - 214**

**Fig 2. Tanglegram of a phylogenetic tree based on the cytochrome c oxidase subunit I (COI) gene of 57 *Culex* (*Cx.*) mosquitoes compared to a hierarchical clustering tree of *Culex* mosquito feeding patterns.** The phylogenetic tree (shown on the left) was generated using IQ-TREE with 1000 ultrafast bootstrap alignments. Node support (bootstrap values) is indicated using coloured circles in the phylogenetic tree. If the node support was less than 50, no circle was placed on the clade. The labels of species that are known vectors for several medically relevant pathogens are bold in the phylogenetic tree (31).The hierarchical clustering tree (shown on the right) was created in R using the *hclust* function. The phylogenetic and feeding tree are connected between the same species. The branch length of the trees represents the distance between clusters. No clusters were found that were the same in both trees. Next to the feeding dendrogram the percentage of feeds on each host group is presented per *Culex* species. The *Chaoboridae* family was chosen as the outgroup. The number of blood-meals and publications per *Culex* species are found in Supporting information 4. For each species the variation in number of blood-meals on major host groups per study is shown in Supporting information 10. The same feeding proportions are lined up with the phylogeny, rather than clustered and rearranged, in Supporting information 11.

The combination of the feeding dendrogram and the tree is confuse, and it is unclear it stastitical power of the dendrogram. e.g. Most Culex pipiens s.l. present higher feeds in birds (with similar proportions of mammals) but they are separated in your representation. Representing the proportion chart in the same order than the phylogentic tree will be more useful to discuss differences between close species;

We thank the reviewer for their suggestion. We have created the suggested figure and included it in Supporting information 11. We have added a reference to this Supporting information in the description of Figure 2.

**Our changes:**

**Discussion – lines 281 - 283**

No relationship between *Culex* phylogeny and feeding patterns was observed. In Supporting information 11, the feeding patterns are directly lined up with the phylogeny, which also shows no clear relationship between feeding and phylogeny.

**Supporting information 11**

Figure 1 Culex phylogeny and the percentage of feeding patterns on 5 major host groups (amphibian, avian, human, non-human mammal, reptile) per Culex species. The phylogenetic tree is based on the cytochrome c oxidase subunit I (COI) gene of 57 Culex (Cx.) mosquitoes. The phylogenetic tree (shown on the left) was generated using IQ-TREE with 1000 ultrafast bootstrap alignments. Node support (=bootstrap values) is indicated using coloured circles in the phylogenetic tree. If the node support was less than 50, no circle was placed on the clade.

I think that you should also represent the variation across studies (or populations) in a plot figure with a median across studies (or populations). This will allow further statistical analysis to verify if the variation observed is statistically different or not (95% confident intervals can be useful for this)

We have analysed the variation across studies per species. This analysis is shown in Supporting information 10. We also analysed the variation across studies between the realms for the three major vectors analysed in Figure 3. This is shown in Supporting information 13.

**Our changes**

**Supporting information 10**

Figure 1 Number of blood-meals taken from each major host group (amphibian, avian, human, non-human mammal, and reptile) for each mosquito species included in this meta-analysis. The title of each graph shows the mosquito species (Number of blood-meals (number of studies)). The red error bars indicate the 95% Confidence interval.

*Figure 1* Feeding patterns of *Culex tritaeniorhynchus* (A), ‘*Culex pipiens* pooled’ (B), and *Culex quinquefasciatus* across different realms. For each realm the total number of blood-meals and total number of publications (blood-meals(publications)) is shown. The red error bars show the 95% Confidence interval. Each dot represents a study.

-line 185: it is unclear the goal for this analysis. Why these groups? the table with the number of studies per species is important to justified this selection; The study is justified due their medical importance for arbovirus transmission but them you cluster the information of Culex pipiens s.l. all together; and lack a proper analysis of all vectors; lack of statistical analysis to discuss this topic. You should define a clear goal and perform a depper analysis with key species.

The groups for the realm analysis were chosen as they had more than a 1000 bloodmeals recorded and they had data available from three or more realms. The number of blood-meals and the number of publications recorded for each species is now available in Supplementary information 4.

-line 220: you should provide further analysis in these species... This may be also clear in the introduction. Maybe label species based on their medical importance in the first table suggested.

We have labelled species of high medical importance in bold in Figure 2. We chose *Culex tritaeniorhynchus, ‘Cx. pipiens* pooled’, and *Cx. quinquefasciatus* as these were amongst the medically important species and were the most well studied with more than a 1000 blood-meals recorded and data in three or more realms.

**Our changes:**

**Results – lines 202 - 214**

**Fig 2. Tanglegram of a phylogenetic tree based on the cytochrome c oxidase subunit I (COI) gene of 57 *Culex* (*Cx.*) mosquitoes compared to a hierarchical clustering tree of *Culex* mosquito feeding patterns.** The phylogenetic tree (shown on the left) was generated using IQ-TREE with 1000 ultrafast bootstrap alignments. Node support (bootstrap values) is indicated using coloured circles in the phylogenetic tree. If the node support was less than 50, no circle was placed on the clade. The labels of species that are known vectors for several medically relevant pathogens are bold in the phylogenetic tree (31).The hierarchical clustering tree (shown on the right) was created in R using the *hclust* function. The phylogenetic and feeding tree are connected between the same species. The branch length of the trees represents the distance between clusters. No clusters were found that were the same in both trees. Next to the feeding dendrogram the percentage of feeds on each host group is presented per *Culex* species. The *Chaoboridae* family was chosen as the outgroup. The number of blood-meals and publications per *Culex* species are found in Supporting information 4. For each species the variation in number of blood-meals on major host groups per study is shown in Supporting information 10. The same feeding proportions are lined up with the phylogeny, rather than clustered and rearranged, in Supporting information 11.

--------------------

**Conclusions**

Reviewer #3: -line 268-272: Domestic animals is also a key factor. The preference of mosquito can also be evaluated by the location of the collection. Gomes et al. 2013 found differences between animal shelters in blood fed females. This type of data may also improve your analysis about the topic (especially for the species with medical importance).

We have now included collection location (indoor/outdoor) and land use type in our analysis. We agree that domestic animals are a key factor in determining mosquito blood-feeding patterns, however most papers do not ask if a domestic animal is nearby.

-Authors should reorganize the discussion after to improve the definition of the hypothesis and the analysis.

Based on the changes in hypotheses and aims made in the introduction we have organised the discussion. The updated discussion is shown below.

**Our changes:**

**Discussion – lines 247 - 375**

**Discussion**

Our analysis of *Culex* feeding patterns revealed that *Culex* mosquitoes have a wide variety of feeding patterns, with some feeding on only mammals, only birds, or only reptiles, but most of them also feeding on multiple host groups. We show that the feeding patterns do not appear to be explained by phylogeny alone. Furthermore, in depth analysis on the highly sampled, ‘*Culex pipiens* pooled’, *Culex quinquefasciatus, and Culex tritaeniorhynchus* in different biogeographical realms showed significantly different feeding patterns. Assessing the feeding patterns of these mosquitoes is essential as they are primary vectors for several important mosquito-borne viruses, including zoonotic viruses (1,2).

*Overall importance of blood-meal analyses*

*Culex* mosquitoes with a wide host range utilization can potentially act as bridge vectors, e.g. for West Nile virus (WNV), transmitting virus from reservoir birds to other susceptible-but-dead-end hosts such as humans and horses. For example, the competent WNV vectors *Culex restuans, Cx. pipiens* (ns)*, Cx. theileri, Cx. quinquefasciatus,* and *Cx. modestus* all had feeding patterns that included both human and avian bloodfeeding (32–35). Other studies have described *Cx. restuans* and *Cx. pipiens* (ns)*.* to be largely ornithophilic (36), which is in line with our results, with a majority of *Cx. restuans* and *Cx. pipiens* (ns) blood-meals being from avian hosts. *Culex quinquefasciatus* is often believed to be a mosquito that can feed on a wide variety of hosts, depending on the abundance of these hosts (37). This is also similar to the results we found as around 34% of all *Cx. quinquefasciatus* feeds was on avian hosts, 17% on humans, 48% on non-human mammals. Very little research has been done on *Cx. modestus* despite this species being a known highly competent vector for WNV in the lab (38). Our results show *Cx. modestus* feeds being 97% on avian hosts. Observations from the UK seem to support high avian and mammalian biting patterns (39). The combination of feeding on both birds and humans emphasizes how this species must not be overlooked as a potential WNV vector. There were several other species which had feeding patterns that included both avian and mammalian hosts but are not well known WNV vectors (*Cx. erythrothorax, Cx. nigripalus, Cx. orbostiensis, Cx. orientalis, Cx. perexiguus, Cx. restrictor, Cx. sitiens*). These species could potentially be implicated in a future outbreak, but perhaps this has not been observed or the species hasn’t been in contact with the virus yet. Thus, being aware of the feeding patterns of mosquito species which are currently not well-known vectors is still relevant to assess future risks.

*Comparison of Culex phylogeny and feeding patterns*

We compared the phylogeny of mosquitoes from the *Culex* genus to their feeding patterns. Hierarchical clustering of *Culex* feeding patterns revealed a different grouping than the clusters of a *Culex* phylogeny based on the *COI* gene. No relationship between *Culex* phylogeny and feeding patterns was observed. In Supporting information 11, the feeding patterns are directly lined up with the phylogeny, which also shows no clear relationship between feeding and phylogeny. This is in line with the results from another meta-analysis which summarized the feeding patterns of Australian mosquitoes (40). However, contrasting results were found in another study looking at the feeding patterns of 256 mosquitoes from multiple genera. Their results indicate that evolutionary relationships and major continental drift events are associated with determining feeding patterns (17). Our results only focus on one genus and recent feeding patterns, and do not show an association between *Culex* evolutionary relationships and host feeding patterns. By focussing on one genus, there is no influence of distantly related genera creating clusters (17). Our genus-specific analysis highlights the differences in feeding patterns at a higher resolution, showing that at genus-level analysis, species clusters do not fully explain feeding patterns.

*Feeding patterns of medically important species across realms*

For three geographically widespread and important vector species, we analysed feeding patterns across different biogeographical realms. For ‘*Cx. pipiens* pooled’, *Cx. quinquefasciatus* and *Cx. tritaeniorhynchus* we observed different host utilizations in different realms. This indicates that the presence of the same species can represent a different risk for different areas. For example, whilst *Cx. quinquefasciatus* might pose a limited threat to humans in the Australasian realm, it could be an important bridge vector in the Afrotropical and Nearctic realm. The reason behind these differences in feeding patterns is unknown, but multiple factors such as host availability, land use, and climate could influence this effect.

*Limitations*

This difference in feeding patterns across realms could be due to a different host availability. Since we did not have data on the availability of host species in each specific study area, we cannot determine whether a high percentage of feeds on a specific host species was due to an actual preference of the mosquito for that host or if it was due to that host species being the most available host compared to other species. A more sophisticated way to measure this, if the data were available, would be by utilizing a forage ratio analysis where the observed frequency of blood-meals on a specific host is divided by the relative abundance of that host in the study area (41). Including an estimate of host availability in future field studies would allow for this analysis of feeding patterns. This could be done using regional host density data where it exists.

Shifts in mosquito feeding behaviour could impact the virus transmission risk to humans. In the US a shift in feeding patterns was observed in *Cx. pipiens* after their main host American robins migrated, which resulted in a switch to humans as their primary host. This increase in *Cx. pipiens* feeding on humans coincided with an increase in people being infected with WNV. A similar pattern was observed for *Cx. tarsalis* where an increased number of human WNV infections occurred if the preferred avian host migrated (44). Thus, as land use change and climate change could drive mosquitoes to feed on different hosts (9,10), this could alter the potential of virus transmission.

Land use could be an important factor in determining host utilization of these mosquitoes. Geographically distinct populations of mosquitoes can have different host preferences (42). Urbanization affects feedings patterns as well, with *Cx. pipiens* and *Cx. quinquefasciatus* having increased levels of human feeding in urban areas (10). Where available we collected the data on the different land use types included in the studies of this meta-analysis (Supporting information 2). We summarized the feeding patterns of each *Culex* species on the major host groups across different land use types in Supporting information 6. Additionally, whether the collection occurs inside or outside can have an effect, with inside collections having a significantly higher percentage of human blood feeding compared to collection that just occurred outside (43). If specified, the collection location (indoor/outdoor) and the collection method was also included in our database as well (Supporting information 2). We analysed the different collection locations and collection method for ‘*Culex pipiens* pooled’, *Culex quinquefasciatus, and Culex tritaeniorhynchus* in Supporting information 7 and 8. These figures show that for each of these species relatively similar collection locations and methods were used. Most of the included studies did include some form of description of land use types, however the descriptions did greatly vary in the amount of detail and often lacked a clear quantification of land-use type. This made it challenging to draw any conclusions from the land use data. Future studies should aim to include a description of land use types in a 1 km radius around the collection point of the bloodfed mosquito. They should also give a clear reason why they classify a certain area as urban, rural, or natural. If the data are not available, studies should provide additional information and/or supplementary pictures of the location of the mosquito-collections. Specific information on whether these collections were performed indoor or outdoor should also be given.

When looking at the differences in feeding patterns between realms there was a large variation in the number of blood-meals recorded in each realm. Additionally, the number of publications available for each species varied greatly. We found a moderate correlation between the number of publications and the number of species, so the countries which had more publications likely had higher numbers of *Culex* species that were included (Supporting information 9). As we did not correct for sampling effort the results might be biased towards feeding patterns that are common in more well sampled areas. Additionally, for some species complexes that are challenging and inconsistent to identify, such as the *Culex pipiens* complex*,* data on individual sub-species/biotype are lacking in many included studies. Consequently, our analyses may not accurately reflect the feeding patterns of lower-order taxa e.g. sub-species for this complex. For most of the studies the local host availability, season, land use, exact time and location of the collected mosquito was not recorded. These details would have given a more comprehensive understanding of the mosquito host feeding patterns. ‘*Culex pipiens* pooled’ showed variation in feeding patterns between realms. The different subtypes contained in this pool have been shown to have different feeding preferences. *Culex pipiens pipiens* and *Culex pipiens molestus* are an example of this, with biotype *pipiens* having a high percentage of avian blood-meals whilst biotype *molestus* had a higher percentage of human blood-meals (45). Thus, the differences in feeding patterns between the realms for ‘*Culex pipiens* pooled’ could have been due to the different *Culex pipiens* subtypes contained in the pool being mostly present in different locations.

*Conclusion*

Analysing the feeding patterns of *Culex* mosquitoes on a global scale showed a large variety in feeding patterns between the *Culex* species and a limited genetic basis for these differences. Additionally, variations in feeding patterns in the different realms for the same species were found, highlighting the importance of local surveillance. The reason behind these different patterns is unknown but indicates extrinsic factors such as host availability, land use and climate playing a large role. When determining how host feeding patterns influence human disease risk a wide variety of external factors should therefore be included in the consideration. Finally, as climate and land use change may alter the feeding patterns on avian and human hosts, there may be an increased potential of mosquito-borne viral epidemics and cross species transmission. There are not enough data available to predict this shift and its possible effects at present, again highlighting the importance of local and periodic surveillance in high-risk areas.

--------------------

**Summary and General Comments**

Reviewer #1: Griep et al. performed a meta-analysis of Culex mosquito feeding patterns. The authors analyzed blood feeding data from 90 publications comprising 26,857 blood meals from 71 different Culex species. For all species, mosquito phylogeny was compared with feeding patterns and for a subset of species a more detailed comparison across biogeographical realms was performed. The authors found that feeding patterns were not significantly explained by mosquito species level phylogeny, and they found variation in feeding patterns between realms. The main conclusions of this study are that feeding patterns vary between Culex species and geographically distinct populations. While the study addresses an important question regarding the feeding patterns of Culex mosquitoes, which is relevant to better understand the risk for pathogen transmission, the overall novelty and significance are limited.

We do believe that the work is novel and significant as we are the first people to show there is no phylogenetic basis for feeding patterns. We were able to do this by doing our analysis at a high resolution focussing on one specific genus instead of comparing between species of multiple genera.

Reviewer #2: This manuscript is a meta-analysis of Culex biting patterns over last 15 years, based on studies that performed molecular analysis of blood meals to the species-level. The topic would be of interest to PLOS NTD readers, given that Culex mosquitoes can serve as vectors for diseases, including those with both animal and human hosts. The authors show that there is no apparent association between genetic relatedness of mosquito species and their preferred blood meal hosts. They also show that within 3 individual species, blood meal hosts differ across biogeographic realms. These results are interesting because they suggest that biting preferences cannot be inferred from Culex species alone, but rather they are specific to local environments. The paper is concise and interesting, however I worry that some major confounders may have been overlooked in the analyses and therefore could threaten the conclusions. Thus, major revisions should be required prior to considering for publication in PLOS NTD.

1. As the authors point out in the discussion, it is well known that the environment surrounding collections (eg indoor/outdoor/urban/rural/natural/etc) can impact the species distribution. It’s hard to imagine that the studies included in these analyses do not provide information about where the collections occurred, and it would be important to know whether the differences in the within-species, across-realm host preferences may be attributable to the environment surrounding collections. Thus, within-species, across-environment comparisons may be valuable for interpreting the findings presented here. Site descriptions are

2. Additionally, how mosquitoes are collected (eg passive trapping vs aspiration vs etc) can impact the species distributions observed. As with environment, the collection methods for the papers analyzed are not discussed and could be similarly driving some of the differences observed. Collection approaches should be reported in the methods sections of the papers analyzed.

3. Regarding the above two points: in a quick check on the first 6 papers in Supplemental Table 1, 5 papers described the site types (eg urban, rural, natural) and collection approaches (eg baited traps, aspiration, etc). Thus, this information should be possible to attain and include in analyses.

We thank the reviewer for their comments. We agree that many other factors such as collection location, land use type, and collection method could affect the results. This is why we have now updated our database to contain all this information. Additionally, we did several additional analyses which can be found in Supporting information 6, 7, 8. These analyses were not included in the main manuscript as these confounding factors can be affected by subjectivity. Definitions of urban, rural, natural can differ between countries and even individual interpretation. This is why we did not make any conclusions of the relation between feeding patterns and land use as these definitions can be highly subjective.

4. Line 103: What was the justification for including zoos? This choice doesn’t seem like it would be representative of a particular biogeographic realm. How do the results look when papers with zoos are removed?

Zoos still represent a real choice for mosquitoes. The realm analyses were performed at higher taxonomic rank so non-native species from different realm have a minimal effect on the overall feeding patterns. Zoos are important to include as this analysis now provides a resource to show what they will bite should the mosquito species or host species be invasive and their ranges overlap in future (which has potentially important medical implications for disease vectors and reservoir/dead end hosts). We have clarified our reasoning to include zoos in the methods.

**Our changes:**

**Methods – lines 107 - 109**

Studies performed in zoos were included, as they are a location where mosquitoes are free to choose or not choose for a specific host under field conditions.

5. Line 157-158/Figure 1: The number of publications and the number of species per geographic location appear to be quite correlated. Can you please quantify this correlation? This should be added to the discussion (Lines 291-292) about sampling effort

We have now included a correlation between the number of publications and number of species in Supporting information 9.

**Our changes:**

**Supporting information 9**

Figure 1 Correlation between the number of publications against the number of Culex species. The circles represent the countries included in this analysis. The R-squared and p value are shown on the right side of the figure.

**Discussion – lines 346 - 351**

Additionally, the number of publications available for each species varied greatly. We found a moderate correlation between the number of publications and the number of species, so the countries which had more publications likely had higher numbers of *Culex* species that were included (Supporting information 9). As we did not correct for sampling effort the results might be biased towards feeding patterns that are common in more well sampled areas

6. Line 173: Is BGK the same thing as the BGI defined in the methods (line 145)?

Thanks for spotting the typo. The abbreviation has now been changed.

**Our changes**

**Results – lines 197 - 198**

The phylogenetic clustering and the feeding clustering showed a very low correlation (BGI = 0.211, Pearson correlation = 0.203) (Fig 2).

7.Lines 196-197: Did you take into account multiple comparisons/df when determining the significance of pairwise comparisons?

No multiple testing correction was applied to the P-values on any test in the paper. As it was unclear from our previous description of the statistical methods, we have moved this description and created a separate paragraph with more details for the analysis of feeding patterns across biogeographical realms. We have clarified the statistical methods in a separate paragraph, see below.

**Our changes**:

**Methods – lines 159 - 163**

*Feeding patterns between realms*

The feeding patterns of three major vectors, *Cx. tritaeniorhynchus, ‘Cx. pipiens* pooled’, and *Cx. quinquefasciatus* were analysed across different biogeographical realms. To assess the difference in feeding patterns between realms Pearson’s chi-square test was used, no multiple testing correction was applied to the P values. This analysis was performed with the stats package (v3.6.2) in R.

8. In general, the notation and legends for Figure 2 need to be clarified and more precise. For example:

a. Lines 201-202: The number of bloodmeals and publications are below the bars

b. Lines 205-207: Are these superscript letters? Or does this refer to the percentages on the bar plots?

The notation for Figure 3 has been updated. Please see the updated version below.

**Our changes:**

**Results – lines 233 – 244**

**Fig 3. Feeding patterns of *‘Culex pipiens* pooled’, *Culex quinquefasciatus*, and *Culex tritaeniorhynchus* in different biogeographical realms.** Percentage of blood-meals on amphibian, avian, human, non-human mammal, and reptile hosts are shown for each realm. The total number of blood-meals and total number of publications (blood-meals(publications)) is shown below each bar. ‘*Culex pipiens* pooled’ consists of the following mosquitoes: *Culex pipiens* not specified (ns)*, Culex pipiens molestus, Culex pipiens pallens, Culex pipiens pipiens, Culex pipiens/molestus* hybrid*.* Significance groups of Chi-square pairwise comparisons are shown in the figure. No multiple corrections were applied to the pairwise comparisons. Superscript letters above the bars indicate significance groups. If two bars share the same letter they do not differ significantly (p>0.05). If two bars share a different letter they do differ significantly (p<0.05). Subscript numbers separate different pairwise comparisons within the three vector species (i.e. there were no pairwise comparisons between different mosquito species). The numbers in the bar represent the percentages of feeds on each host group for a specific species in that realm. See Supporting information 12 for the full list of pairwise comparisons and P values. The variation between studies for this analysis is shown in Supporting information 13.

9. Lines 220-222: Do the WNV-competent vectors include avian and human hosts across all biogeographical realms?

Yes, as a higher percentage of human biting there is a higher risk

10. Lines 229-230: I’m not sure that unpublished results are appropriate for a meta-analysis, but at a minimum, it would be good to have some sort of quantitation on this statement about high human biting behavior of Cx. modestus, and perhaps some speculation on why the UK results are different from the results of your paper.

We have found an updated reference and now have included this in the paper.

**Our changes:**

**Discussion – lines 268 – 269**

Observations from the UK seem to support high avian and mammalian biting patterns (39).

11. Lines 248-249: You mention here that Cx. tritaeniorhynchus had similar feeding patters across realms, but Fig 2 and Supplemental table 4 indicate that host distributions were different across realms.

Thank review for spotting this mistake. We have removed this error.

**Our changes:**

**Discussion – lines 249 - 252**

We show that the feeding patterns do not appear to be explained by phylogeny alone. For ‘*Culex pipiens* pooled’, *Culex quinquefasciatus, and Culex tritaeniorhynchus* different biogeographical realms showed significantly different feeding patterns.

12. Lines 273-278: As stated above, most studies likely describe how and where collections occur.

We have added more detailed information on collection location (indoor/outdoor), collection method, and land use type in our database. We have analysed the additional data that was added to our database and the figures are included in Supporting information 6, 7, 8.

**Our changes:**

**Methods – lines 114 – 119**

From the articles that were included the following information was extracted: PMID, Title, Authors, Publication year, DOI, Mosquito species, Mosquito location (country), Biogeographical realm, Host (to level described in literature), Host (major host group: amphibian, avian, human, non-human mammalian, and reptilian), Number of bloodmeals, Total number of bloodmeals (in that specific study), Collection method, Bloodmeal analysis method, Land use type (of where study was done), Indoor/outdoor collections.

**Methods – lines 165 - 169**

*Study characteristics*

For every included *Culex* species the percentage of feeds across different land use types was analysed. These results are described in Supporting information 6. For *Cx. tritaeniorhynchus, ‘Cx. pipiens* pooled’, and *Cx. quinquefasciatus* the different collection methods and collection locations (indoor/outdoor) were analysed as well. These results are presented in Supporting information 7 and 8 respectively.

**Discussion – lines 323 - 343**

Land use could be an important factor in determining host utilization of these mosquitoes. Geographically distinct populations of mosquitoes can have different host preferences (42). Urbanization affects feedings patterns as well, with Cx. pipiens and Cx. quinquefasciatus having increased levels of human feeding in urban areas (10). Where available we collected the data on the different land use types included in the studies of this meta-analysis (Supporting information 2). We summarized the feeding patterns of each Culex species on the major host groups across different land use types in Supporting information 6. Additionally, whether the collection occurs inside or outside can have an effect, with inside collections having a significantly higher percentage of human blood feeding compared to collection that just occurred outside (43). If specified, the collection location (indoor/outdoor) and the collection method was also included in our database as well (Supporting information 2). We analysed the different collection locations and collection method for ‘Culex pipiens pooled’, Culex quinquefasciatus, and Culex tritaeniorhynchus in Supporting information 7 and 8. These figures show that for each of these species relatively similar collection locations and methods were used. Most of the included studies did include some form of description of land use types, however the descriptions did greatly vary in the amount of detail and often lacked a clear quantification of land-use type. This made it challenging to draw any conclusions from the land use data. Future studies should aim to include a description of land use types in a 1 km radius around the collection point of the bloodfed mosquito. They should also give a clear reason why they classify a certain area as urban, rural, or natural. If the data are not available, studies should provide additional information and/or supplementary pictures of the location of the mosquito-collections. Specific information on whether these collections were performed indoor or outdoor should also be given.

**Supporting information 6**

Figure 1 *Culex* feeding patterns across different land use types. Each figure shows the percentage of blood-meals taken on five major host groups: amphibian, avian, human, non-human mammal, reptile per mosquito species. Where data was available land use from different studies was classified into six different types: mixed (mosquitoes from multiple types of land use were collected in this study), natural (land that is not modified for human use, such as wetlands, forests, grasslands, etc.), not specified (if no detailed description was given of land use the study was classified as not specified), rural (if this was described in the original publication, or the publication described low population density, small settlements, farming, and agriculture), urban (if this was described in the original publication, or the publication described a major city), and zoo. The title of each graph shows the mosquito species (Number of blood-meals (number of studies)).

**Supporting information 7**

Figure 1 Percentage of blood-meals per major host group: amphibian, avian, human, non-human mammal, and reptile, that were collected using different methods, or combinations of methods. This analysis was done for ‘Culex pipiens pooled’, Culex quinquefasciatus, Culex tritaeniorhynchus. ‘Culex pipiens pooled’ describes a pool of species that contains: Culex pipiens not specified (ns), Culex pipiens molestus, Culex pipiens pallens, Culex pipiens pipiens, Culex pipiens/molestus hybrid. The title of each graph shows the mosquito species (Number of blood-meals (number of studies)). In each bar the exact number of blood-meals collected using the different methods is shown.

**Supporting information 8**

Figure 1 Percentage of blood-meals collected indoor, mixed (=indoor and outdoor), not specified, and outdoor per major host group: amphibian, avian, human, non-human mammal, and reptile. This analysis was done for ‘Culex pipiens pooled’, Culex quinquefasciatus, Culex tritaeniorhynchus. ‘Culex pipiens pooled’ describes a pool of species that contains: Culex pipiens not specified (ns), Culex pipiens molestus, Culex pipiens pallens, Culex pipiens pipiens, Culex pipiens/molestus hybrid. The title of each graph shows the mosquito species (Number of blood-meals (number of studies)). In each bar the number of blood-meals is shown for each collection location.

Reviewer #3: The manuscript approuch an interest topic for mosquito biology, but authors should improve how they present the information and define better the goals fo the manuscript.

We have changed our introduction to better describe the goals for the manuscript.

**Introduction – lines 85 - 92**

Here, we analyse the data published over the last 15 years describing *Culex* blood-feeding in the field. We first look at the whole genus level and then focus in on three important *Culex* vectors. For the whole *Culex* genus we analyse whether there is a pattern between phylogenetic relatedness and feeding patterns. For three important vector species we assess the relationship between biogeographical realm and feeding patterns. Overall, this analysis enables a global insight into the feeding patterns of *Culex* mosquitoes and thus provides higher-level data for risk assessments to specific hosts, and to specific regions, which will inform ongoing efforts, particularly in Europe (18,19), to mitigate the spread of *Culex*-borne viruses (e.g. West Nile virus and Usutu virus) (20).
